# Traumatic Brain Injury Preserves Firing Rates But Disrupts Laminar Oscillatory Coupling and Neuronal Entrainment in Hippocampal CA1

**DOI:** 10.1523/ENEURO.0495-19.2020

**Published:** 2020-09-02

**Authors:** Paul F. Koch, Carlo Cottone, Christopher D. Adam, Alexandra V. Ulyanova, Robin J. Russo, Maura T. Weber, John D. Arena, Victoria E. Johnson, John A. Wolf

**Affiliations:** 1 Center for Brain Injury and Repair, Department of Neurosurgery, University of Pennsylvania, Philadelphia, Pennsylvania 19104; 2 Corporal Michael J. Crescenz Veterans Affairs Medical Center, Philadelphia, Pennsylvania 19104

**Keywords:** hippocampus, medial septum, oscillations rodents, synchronization, TBI

## Abstract

While hippocampal-dependent learning and memory are particularly vulnerable to traumatic brain injury (TBI), the functional status of individual hippocampal neurons and their interactions with oscillations are unknown following injury. Using the most common rodent TBI model and laminar recordings in CA1, we found a significant reduction in oscillatory input into the radiatum layer of CA1 after TBI. Surprisingly, CA1 neurons maintained normal firing rates despite attenuated input, but did not maintain appropriate synchronization with this oscillatory input or with local high-frequency oscillations. Normal synchronization between these coordinating oscillations was also impaired. Simultaneous recordings of medial septal neurons known to participate in theta oscillations revealed increased GABAergic/glutamatergic firing rates postinjury under anesthesia, potentially because of a loss of modulating feedback from the hippocampus. These results suggest that TBI leads to a profound disruption of connectivity and oscillatory interactions, potentially disrupting the timing of CA1 neuronal ensembles that underlie aspects of learning and memory.

## Significance Statement

The hippocampus and its connections have been hypothesized to be particularly vulnerable to traumatic brain injury (TBI) and therefore are implicated in post-TBI memory impairments. However, it remains an open question whether loss of neurons, their activity, or an encoding disruption underlies such deficits, and whether the remaining neurons are capable of recovering the function of these networks. Using laminar silicon probes spanning the entire CA1 subregion, this study provides the first *in vivo* evidence that hippocampal CA1 single unit activity post-TBI can maintain a normal firing rate despite significantly attenuated, layer-specific loss of input. They cannot, however, maintain normal synchronization to the dominant oscillations within the hippocampus, a critical component of hippocampal memory encoding and decoding mechanisms.

## Introduction

Learning and memory impairments are a common and debilitating consequence of traumatic brain injury (TBI) for which there are few effective treatments, presenting a significant burden to society (Capruso and Levin, 1992; Monti et al., 2013; Maas et al., 2017; Wilson et al., 2017). While the neuropathologies of TBI are diverse and heterogeneous, the hippocampus (HC) and its connections have been reported as highly vulnerable to traumatic injury both in humans and multiple experimental TBI models (Smith et al., 1991, 1997; Hicks et al., 1993; Blumbergs et al., 1995; Toth et al., 1997; Grady et al., 2003). Moreover, impairment of learning and memory tasks following experimental TBI has long been observed in association with hippocampal pathology and changes in hippocampal circuitry, largely through *ex vivo* investigations in slice preparations (Reeves et al., 1997; Howard et al., 2007; Norris and Scheff, 2009; Almeida-Suhett et al., 2015; Wolf et al., 2017). Comparatively little evidence has been reported describing alterations in hippocampal network-level neuronal activity patterns in the intact brain that are thought to support the ability to encode and decode information. These disruptions may underlie hippocampal-dependent memory deficits repeatedly observed after experimental TBI (Smith et al., 1997).

Synchronized hippocampal synaptic activity can be observed as distinct, organized oscillations within the local field potential (LFP), each with unique signatures that include preferred frequency bands as well as patterns of power and phase at different depths within the anatomic organization of the hippocampal layers. These oscillations are crucial for hippocampal-dependent memory performance, and include theta, gamma, and sharp wave-associated ripple (SPW-R) oscillations (O’Keefe and Recce, 1993; Foster and Wilson, 2006; Lisman and Buzsáki, 2008; Buzsáki, 2015). Each of these represent different patterns of coordinated synaptic input and can drive the firing patterns of local hippocampal pyramidal cells and interneurons depending on the current network and behavioral state. The relationship between coordinated synaptic input into the hippocampus (observed as LFP oscillations and waves), and the timing of local neuronal activity is an important component of information encoding, recall, and, potentially, consolidation (Carr et al., 2011; Buzsáki and Moser, 2013). Whether coordination of synaptic inputs at the hippocampal network level or neuronal firing patterns driven by these inputs become disrupted after TBI is unknown.

Several reports from animal models have described a loss of hippocampal theta power after TBI that correlated with spatial memory deficits, as well as early evidence that the exogenous restoration of theta may rescue memory performance (Fedor et al., 2010; Lee et al., 2013, 2015). However, others have found only a nonspecific, broadband reduction in hippocampal oscillatory power after injury (Paterno et al., 2016). It is unknown whether alterations in hippocampal oscillatory power after TBI reflect changes in specific synchronized synaptic inputs and lead to a change in hippocampal neuronal firing patterns, or whether power losses are nonspecific and potentially related to overall reductions in local neuron firing rates. We therefore used high-density silicon probes to record both the LFP and local neuron activity at multiple depths simultaneously across the anatomic laminar structure of CA1. This methodology allowed us to localize activity to specific layers (allowing identification of specific afferent projections into CA1) as well as to explore the relationship between synchronized synaptic input and local neuronal firing patterns after TBI. Given that the medial septum (MS) is implicated in the generation and maintenance of theta oscillations (Müller and Remy, 2018), we also explored whether communication between the hippocampus and the reciprocally connected medial septum was altered after TBI.

At 1 week following the most common rodent model of TBI, we found frequency and anatomic layer-specific losses of oscillatory power in CA1 under anesthesia. Low-frequency power losses were consistent with reduced or disorganized synaptic input into stratum radiatum (SR), but preserved theta oscillations, while high-frequency oscillatory power losses were restricted to the pyramidal cell layer. Single CA1 neurons maintained normal firing rates in the face of attenuated input after TBI, revealing that a subset of CA1 neurons can reach normal firing capacity. However, their ability to properly synchronize to dominant synaptic input and to local high-frequency oscillations was impaired. Single GABAergic medial septal neurons demonstrated increased firing rates after TBI and a reduced ability to synchronize to CA1 synaptic input, despite preserved synchronization with CA1 theta oscillations, suggesting impaired CA1 feedback. These findings support the hypothesis that oscillatory power losses after TBI reflect disruptions in anatomically specific synaptic inputs into the hippocampus, potentially affecting the organization of CA1 ensembles that underlie aspects of learning and memory dysfunction following TBI.

## Materials and Methods

Details of resources used in this study are provided in Table 1.

### Animal care

All animal procedures were performed in accordance with the regulations of the University of Pennsylvania animal care committee. Young, healthy, adult male Sprague Dawley rats (RGD catalog #734476, Charles River Laboratories; RRID:RGD_734476) naive to any previous procedures or investigations were used in this study. Rats were chosen based on weight (range, 345–425 g, median weight, 383.5 g). Animals were initially pair housed but were subsequently housed singly after the injury or sham procedure. Rats were provided standard rat chow and water *ad libitum*. They were kept on a strict reversed 12 h light/dark cycle. After injury, or sham surgery, animals were examined daily for signs of pain or distress.

**Table 1:** Key resources

Reagent or resource	Source	Identifier
Antibodies		
N-terminal amino acids 66–81 of theAPP, Clone 22C11	Millipore	Catalog #MAB348, Millipore;RRID: AB_94882
Ab246, the caspase-derived fragment ofα-spectrin	Courtesy of Dr. Robert Siman, Universityof Pennsylvania	Oo et al. (2002)
Chemicals, peptides, and recombinantproteins		
Ketamine	West-Ward	Catalog #NDC0143-9509-01
Xylazine	Akorn	Catalog #NDC59399-110-20
Acepromazine	Clipper Distributing Company	Catalog #NDC57319-604-04
Normal horse serum	Vector Laboratories	Catalog #S-2000-20
Fluoro-Jade C	Sigma-Aldrich	Catalog #AG325
Bupivacaine	Medline Industries	Catalog #0409-1610-50
Glycopyrrolate	Hikma Pharmaceuticals	Catalog #NDC0143-9682-01
Screw, size 2-56x3/8’’	Small Parts	Catalog #MPX-0256-06F-M
Luer Lock hub	Becton Dickinson	Catalog #305165
Dentsply Caulk Orthodontic Resin(powder, liquid)	BencoDental	Catalog #1007-910, catalog #1007-894
Low-toxicity silicon adhesive, Kwik-Sil	World Precision Instruments	Catalog #KWIK-SIL
A silk suture, 4/0RB1 30'	Ethicon US	Catalog #K871H
Buprenex	Par Pharmaceuticals	Catalog #NDC 42023-179-01
Tungsten electrode	FHC	Catalog #UEWSEGSEBNNM
32-channel silicon probe	NeuroNexus Technologies	Catalog #A1X32-Poly2-5mm-50s-177-H32
Thomas tetrode electrode	Thomas RECORDING	Catalog #AN000020
Optimax buffer	BioGenex	Catalog #HK583-5KE
Experimental models: organisms/strains		
Sprague Dawley rats	Charles River Laboratories	Catalog #734476; RRID:RGD_734476
Software and algorithms		
FP302	http://amscien.com/AmsFluid.htm Instruments	
MATLAB, version R2017a	MathWorks	MATLAB; RRID:SCR_001622
FMAToolbox	http://fmatoolbox.sourceforge.net/	FMAToolbox; RRID:SCR_015533
Chronux	http://chronux.org	Chronux; RRID:SCR_005547
CSDPlotter, version 0.1.1	https://github.com/espenhgn/CSDplotter	Pettersen et al. (2006)
KlustaKwik	http://klusta-team.github.io/klustakwik/	KlustaKwik; RRID:SCR_014480
Circular statistics	https://es.mathworks.com/matlabcentral/fileexchange/10676-circular-statistics-toolbox-directional-statistics	Circular statistics; RRID:SCR_016651
GraphPad Prism	http://www.graphpad.com/	GraphPad Prism; RRID:SCR_002798
KlustaViewa	https://github.com/klusta-team/klustaviewa	
phy	https://github.com/kwikteam/phy	
Noldus Information Technology	https://www.noldus.com	Noldus; RRID:SCR_004074
EthoVision XT automated trackingsoftware	https://www.noldus.com/ethovision-xt	EthoVision XT; RRID:SCR_000441

### Experimental design

Twenty-one rats underwent initial training on the Morris water maze (MWM) in two sessions 24 h before being randomized to undergo fluid percussion injury (FPI) or sham surgery. Rats found to have a dural breach or a subdural hematoma at the time of surgery were excluded. On postsurgery day 1, rats underwent memory testing on the Morris water maze. On postsurgery day 7, five of the injured and five of the sham-operated rats were randomized to undergo acute electrophysiological recording under anesthesia followed by sacrifice. An additional 10 rats (five injured, five sham) were sacrificed on postsurgery day 7 for histologic examination without undergoing electrophysiological recordings. Behavioral testing, electrophysiological recordings, histologic analysis, and initial data processing and spike sorting were performed blinded to the group status of each rat.

### Morris water maze

The MWM is a widely used test of spatial learning and memory (Vorhees and Williams, 2006). We chose to implement a particular MWM protocol designed to specifically test hippocampal-dependent spatial memory, as distinct from broader learning/memory paradigms, and which has been demonstrated to be sensitive to the lateral fluid percussion injury (Smith et al., 1991). The apparatus consisted of an aluminum and Plexiglas circular pool 2 m in diameter and 50 cm tall with the inner surfaces painted black. The pool was filled with 18°C water to a depth of 25 cm. A black 11.5 × 11.5 cm platform that is 24 cm tall was placed in a fixed location within the pool. The black color of the platform and interior of the pool made the platform invisible from the surface. The pool was contained within a room with various objects placed within it, easily seen from within the pool. These objects remain fixed throughout training and testing to provide external visual cues for the rats. A video camera was placed above the pool and an automated video tracking system (EthoVision XT, Noldus; RRID:SCR_000441) recorded the position of the rat.

#### Preinjury training

Rats were selected that had no signs of ocular defect or any other health concerns. Animals were placed in the pool at a random location against the side of the tank and then allowed to freely swim, with the idea that they can escape the water by finding the invisible submerged platform. On the afternoon of the day before injury or sham injury, each animal was placed in the pool and given 2 min to find the platform. If an animal did not find the platform within the 2 min, they were placed on the platform and allowed to remain for 30 s on the initial trial and 15 s on subsequent trials. The starting location for each trial was randomly chosen from four positions situated 90° apart. A total of 10 trials were performed for each rat during the initial session, and the times taken to find the platform (latency) were recorded. Animals were then returned to their home cages to rest overnight. On the morning of injury or sham surgery, each rat underwent an additional 10 trials in an identical manner.

#### Postinjury testing

Approximately 42 h after undergoing fluid percussion injury or sham surgery, surviving rats were then tested for platform memory in the pool. After removing the submerged platform, each animal was released in the pool and the swimming activity of the rat was recorded for two trials of 1 min each. Using the automated tracking software EthoVision XT (RRID:SCR_000441) by Noldus (RRID:SCR_004074), a virtual grid over the surface of the pool was constructed, consisting of five zones of increasing distances from the former position of the platform. Time to first crossing of the platform position was calculated as the latency time. A memory score was also calculated, as detailed by Smith et al. (1991). Briefly, time spent in zones closer to the platform position were weighted higher than time spent in zones further away. These weighted times were then summed to give a memory score. Higher scores indicate better memory performance.

### Fluid percussion injury and sham surgery

At 2 h after the completion of MWM training, animals were weighed and anesthetized with 5% isoflurane. After being placed in a stereotaxic head holder and maintained at 3% isoflurane, ophthalmic ointment was applied to the eyes for vision protection during surgery. Body temperature was maintained with the use of a heating pad throughout the surgery. Subcutaneous injections of bupivacaine (2 mg/kg; catalog #0409-1610-50, Medline Industries) and glycopyrrolate (0.01 mg/kg; catalog #NDC 0143-9682-01, Hikma Pharmaceuticals) were administered followed by a midline incision and skull exposure. Next, a 5-mm-diameter craniectomy was performed over the left parietal cortex midway between bregma and lambda, with the center ∼2.5 mm lateral from midline, centering the defect between midline and the temporal ridge. Rats were maintained at 2–2.5% isoflurane. A screw (size, 2–56 × 3/8 inch; catalog #MPX-0256-06F-M, Small Parts) was inserted anterior to bregma on the right hemisphere for additional structural support. Both FPI-injured and sham animals received a craniotomy and drill hole. Next, a Luer Lock hub (from a 21 gauge needle; catalog #305165, Becton Dickinson) was secured to the craniotomy site with super glue (All Purpose Krazy Glue No Run Gel, Elmer’s Products) followed by the application of dental cement [Dentsply Caulk Orthodontic Resin; catalog #1007-910 (powder) and catalog #1007-894 (liquid), BencoDental] to ensure the formation of a secure head cap before injury. The FPI device (AmScien Instruments) consisted of a Plexiglas, cylindrical, saline-filled reservoir bounded at one end by a Plexiglas plunger mounted on O-rings. The opposite end of the cylinder was capped with a male luer stub, which connected to the Luer Lock cemented on the craniotomy. After removal of anesthesia, animals were monitored for increased respiration, at which point they were attached to the device. For the injury group, once secured, a pendulum was dropped from 20°, striking the plunger and causing a rapid and high-pressure injection of saline into the closed cranial cavity, compressing the brain. The injury level was measured in atmospheres (atm; FPI = 2.3 ± 0.1 atm, mean ± SD) by a pressure transducer, and both the injury trace and atmospheres were recorded (catalog #FP302, AmScien Instruments). For the sham surgery group, the pendulum was not dropped. Animals were then monitored for apnea and seizure postinjury and once stabilized were placed on 1% isoflurane. Before suturing, a low-toxicity silicon adhesive (catalog #KWIK-SIL, World Precision Instruments) was applied to the craniotomy site to mitigate scarring, after which the incision was closed with a silk suture (4/0 RB1 30'; catalog #K871H, Ethicon US). Animals were recovered under a heat lamp and administered a subcutaneous injection of Buprenex (0.05 mg/kg; catalog #NDC 42 023-179-01, Par Pharmaceuticals) after demonstrating sufficient recovery of the righting reflex.

### Acute recording procedure

On postinjury or post-sham surgery day 7 animals were anesthetized in a Plexiglas chamber with 5% isoflurane. They were then placed in a stereotaxic frame using standard ear bars (Stoelting), and the head was leveled under 3% isoflurane. Ophthalmic ointment was applied to the eyes for protection of the cornea during surgery. Body temperature was maintained with the use of a heating pad throughout the surgery. Periodic tail pinches were performed throughout the recording session to ensure an adequate depth of anesthesia. Subcutaneous injections of bupivacaine (2 mg/kg; catalog #0409-1610-50, Medline Industries) and glycopyrrolate (0.01 mg/kg; catalog #NDC 0143-9682-01, Hikma Pharmaceuticals) were administered. The skull was then exposed and leveled with respect to bregma and lambda suture landmarks per the stereotaxic methods of Paxinos and Watson (2007). A skull screw was placed over the right cerebellum to serve as an animal ground. A tungsten electrode (Ø, 125 μm; length, 60 mm; impedance, 0.5 MΩ; catalog #UEWSEGSEBNNM, FHC) with the tip clipped and stripped was inserted into the right lateral ventricle [anteroposterior (AP), 2.0; ML, 1.6; DV, 3.45] to serve as an intracranial reference signal. We then expanded the previously created craniectomy over the left frontoparietal cortex and created a second craniectomy crossing midline, eccentric to the left and just anterior to bregma. A 32-channel silicon probe (catalog #A1X32-Poly2-5 mm-50s-177-H32, NeuroNexus) was then slowly inserted into the left dorsal hippocampus (AP, −4.2; ML, 2.7). Signals from all 32 channels were amplified and visualized in real time during insertion and monitored both for changes in the LFP and spiking activity. Once spiking activity representing the pyramidal cell layer of CA1 was identified on the distal-most channels, this activity was followed proximally along the channels of the probe as it was inserted deeper until it was detected in the proximal third of the probe (final depth range, 2.73–4.19 mm; median, 3.19 mm). The isoflurane was then lightened to the minimum dose required for no response from a tail pinch (range, 1.5-2). After a 30 min period, during which isoflurane levels and signals were allowed to reach a steady state, a 15 min recording was performed. A tetrode (catalog #AN000020, Thomas RECORDING) was then inserted into the left medial septum (AP, 0.6; ML, 0.5; lateral to medial angle of 5°). Spiking activity was monitored, and 5 min recordings were made at each depth below 5.2 mm at which spiking activity could be isolated (minimum of 100 μm separation).

At the end of the electrophysiological recording procedure, the level of isoflurane anesthesia was raised to 5%. After checking for pain responses, the animal was sacrificed by injecting ketamine (75 mg/kg; catalog #NDC 0143-9509-01, West-Ward), xylazine (15 mg/kg; catalog #NDC 59 399-110-20, Akorn), and acepromazine (2 mg/kg; catalog #NDC 57 319-604-04, Clipper). The animal underwent transcardial perfusion with 0.9% heparinized saline followed by 10% neutral buffered formalin (NBF), and the brain was extracted in a manner identical to that in the nonelectrophysiology cohorts (see Histologic examinations section).

### Data acquisition

All electrophysiological recordings were made under isoflurane anesthesia. Neural signals were amplified and acquired continuously at 32 kHz on a 64-channel Digital Lynx 4SX acquisition system (Neuralynx). Raw signals were filtered from 0.1 Hz to 9 kHz on the acquisition board, digitized, and saved. The filtered signal was also displayed for real-time inspection of the LFP during recordings. The raw signals were bandpass filtered from 600 to 6000 Hz for real-time spike inspection. Spike thresholds were manually adjusted during recordings for real-time display purposes based on observed signal-to-noise ratios during the recording session.

### Histologic examinations

A separate subset of animals underwent FPI (*n* = 4) or sham surgery (*n* = 5) only (no electrophysiology) to permit neuropathological examination of the model without the confounding pathology caused by electrode implantation. All animals survived for 7 d and were otherwise handled under protocols identical to those animals that underwent electrophysiological examinations. At the study endpoint, a mixture of ketamine (75 mg/kg; catalog #NDC 0143-9509-01, West-Ward), xylazine (15 mg/kg; catalog #NDC 59 399-110-20, Akorn), and acepromazine (2 mg/kg; catalog #NDC 57 319-604-04, Clipper) was administered. Following adequate anesthesia, animals were transcardially perfused with an initial flush of chilled and heparinized 1× PBS followed immediately with 200 ml of chilled 10% NBF. Brains were extracted and postfixed for 24 h at 4°C before being blocked in the coronal plane at 2 mm intervals and processed to paraffin using standard techniques. Eight-micrometer-thick sections were obtained every 2 mm throughout the rostrocaudal extent of the brain using a rotary microtome. To permit characterization and verify reproducibility of the injury model, tissue sections were first stained with hematoxylin and eosin (H&E) as described previously (Johnson et al., 2013a).

#### Immunohistochemistry

To examine for the presence and distribution of axonal pathology and cell death, standard immunohistochemistry (IHC) techniques were performed as previously described (Johnson et al., 2013a, 2016, 2018). Briefly, following deparaffinization and rehydration of tissue sections, endogenous peroxidase activity tissue was quenched using 3% aqueous hydrogen peroxide (15 min). Antigen retrieval was performed using a microwave pressure cooker with sections immersed in Tris EDTA buffer, pH 8.0. Subsequent blocking was performed for 30 min in 1% normal horse serum (Vector Laboratories) in Optimax buffer (BioGenex). Incubation with the primary antibodies was performed at 4°C overnight. Specifically, to identify axonal pathology, sections were labeled with an antibody reactive for the N-terminal amino acids 66–81 of the amyloid precursor protein (APP; 1:130,000; Clone 22C11, Millipore; Gentleman et al., 1993; Johnson et al., 2016) and Ab246 specific for the caspase-derived fragment of α-spectrin, indicative of apoptosis (1:17,000; Bakshi et al., 2005; Chen et al., 2005; Oo et al., 2002; courtesy of Robert Siman, University of Pennsylvania). After rinsing, sections were incubated with the appropriate biotinylated secondary antibody for 30 min followed by avidin–biotin complex and visualization achieved using DAB (all reagents are from Vector Laboratories). Sections were rinsed and dehydrated in graded alcohols and cleared in xylenes before being coverslipped. Positive control tissue for APP IHC included sections of contused rat tissue with previously established axonal pathology. Omission of the primary antibodies was performed on positive control tissue to control for nonspecific binding.

#### Fluoro-Jade C staining

As an additional marker of degenerating neurons, Fluoro-Jade C staining was performed (Schmued et al., 2005). Specifically, following dewaxing and rehydration to water as above, tissue was the immersed in a 0.06% potassium permanganate solution for 20 min at room temperature. After being rinsed in gently flowing distilled H_2_O, tissue was then incubated in 0.00012% Fluoro-Jade C solution (in 0.1% acetic acid) at room temperature for 30 min. After rinsing, sections were dried at 37°C for 90 min before being immersed in xylenes and coverslipped as above.

#### Analysis of histologic findings

Axonal pathology was examined within a tissue section at the level of the hippocampus (AP, −2.76) and included specific assessment of the fimbria fornix, angular bundle, stratum radiatum, stratum lacunosum-moleculare (SL-M), and stratum oriens (SO) of the hippocampus. The medial septum was also examined at AP +0.60. In addition, cell death was assessed by examination for Fluoro-Jade C and Ab246-positive cells in the medial septum, CA1 and CA3 subfields, and stratum oriens of the hippocampus. The presence or absence of pathology was recorded. A high level of inter-rater reliability in the identification of pathology by two independent observers was achieved (Cohen’s kappa, 0.98).

### Statistics

All statistical analyses were performed in Prism 7 or 8 (GraphPad Prism; RRID:SCR_002798) or using the MATLAB Circular Statistics Toolbox (circular statistics; RRID:SCR_016651). Samples were reported as either numbers of rats or numbers of cells. When samples were numbers of rats, we reported the median and range. When samples were numbers of cells, we reported means and SDs. The Welch’s *t* test, or Mann–Whitney test was used for mean or median group comparisons, respectively. All plots with error bars depict the mean ± SD or median with interquartile ranges, unless otherwise indicated. In the case of LFP power comparisons between groups, the repeated-measures ANOVA was used, and plots with error bars of these data are reported as the mean ± SEM. In the cases of comparing modulation index (MI) values between groups (phase amplitude coupling and single-unit entrainment to high-frequency oscillations), comparisons were made using the Student’s *t* test and plots with error bars of these data are reported as the mean ± SEM.

### Electrophysiology data analysis

Raw signals recorded with the 32-channel silicon probe were downsampled to 2 kHz for LFP analysis. Signals were imported into MATLAB software, version R2017a (MATLAB; RRID:SCR_001622) and processed using a combination of custom and modified routines from the freely available MATLAB packages FMAToolbox (FMAToolbox; RRID:SCR_015533) and Chronux (RRID:SCR_005547; Hazan et al., 2006; Mitra and Bokil, 2008).

#### Channel alignment

For comparative analysis of neurophysiological signals between the injured and sham groups of animals, we first bandpass filtered (0.6–6 kHz) the LFP for each channel of the 32-channel HC probe, giving an estimate of the multiunit spiking activity. We then selected the channel with the maximum average power for each animal and designated that channel to be the pyramidal cell layer. HC probes from all animals were then aligned to this channel. This procedure resulted in 25 aligned channels of neurophysiological data common to all animals.

#### Current source density

Current source density (CSD) of the LFP across all HC channels was calculated with the spline inverse CSD method of Pettersen et al. (2006), using the freely available MATLAB package CSDplotter version 0.1.1 (https://github.com/espenhgn/CSDplotter) visualized by convention as a heat map of current sinks (blue) and sources (orange; Mitzdorf, 1985).

#### LFP power

To investigate any statistical differences in power between the two groups and among each of the 25 channels, we windowed each 15 min recording in 1 s epochs and calculated the power spectral density in the 1–300 Hz frequency band (Welch's method: frequency resolution, 0.5 Hz; overlap, 60%) for each epoch. As a result, we obtained a 25 (channels) × 600 (frequency bins) × 2250 (epochs) matrix of power values for each subject. We then calculated the channel–frequency map power difference distribution (PDD) relative to the sham group [(injured/sham − 1)%], averaging the aforementioned matrix along the epochs and groups. To evaluate where these differences were statistically significant, we calculated a significance mask through a repeated-measures ANOVA between the two groups using the epochs as independent variables, obtaining a channel–frequency map of *p* values: *p* values <0.05 were considered significant and converted to a value of 1, otherwise values were represented with a 0. Finally, we multiplied the significance mask by the PDD to identify where the two groups displayed statistically significant differences.

#### Phase amplitude coupling

To investigate the interaction between oscillations in different frequency bands after injury, we used phase–amplitude coupling (PAC) as a form of cross-frequency coupling analysis in the HC. We investigated how the phase of lower-frequency oscillations drive the power of coupled higher-frequency oscillations by measuring the degree of synchronization of the amplitude envelope of faster rhythms with the phase of slower rhythms. To quantify the PAC between strata radiatum and pyramidale LFP, we used the Kullback–Leibler (KL)-based MI based on the joint entropy (Tort et al., 2010).

Briefly, MI was calculated as follows: components of the stratum pyramidale (SP) LFP for each frequency [1–300 Hz, denoted as $\text{Pyr}\left(t,f_{\text{pyr}}\right),f_{\text{pyr}}=1,2,3\text{...}299,300\;\text{Hz}$] were extracted by wavelet transform (Morlet; 10 cycles). The phases of the bandpass filtered (1–20 Hz) stratum radiatum LFP (denoted as $\Phi_{\text{Rad}\left({\text{t,f}}_{\text{rad}}\right)},f_{\text{rad}}=1,2,3\text{...}20\;\text{Hz}$) were obtained from the standard Hilbert transform. The Hilbert transform was also applied to extract time series of the amplitude envelopes (denoted as $A_{\text{Pyr(}t\text{,}f_{\text{pyr}}\text{)}}$) of $\text{Pyr}\left(t,f_{\text{pyr}}\right)$. The composite time–frequency series ($\Phi_{\text{Rad}\left(t,f_{\text{rad}}\right)}$, $A_{\text{Pyr}(t,f_{\text{pyr}})}$) was then constructed, which gave the amplitude of the pyramidal layer LFP for each frequency bin oscillation (1–300 Hz) at each phase of the radiatum layer LFP for each frequency bin (1–20 Hz). Next, the phases $\Phi_{\text{Rad}\left(t,f_{\text{rad}}\right)}$ were binned and the mean value of $A_{\text{Pyr}(t,f_{\text{pyr}})}$ over each binned phase was calculated. We denoted $\left[A_{\text{Pyr}(t,f_{\text{pyr}})}]\Phi_{\text{Rad}\left(t,f_{\text{rad}}\right)}(j\right)$ for the mean $A_{\text{Pyr}(t,f_{\text{pyr}})}$ value at the phase bin *j*. Afterward, the normalized mean amplitude of $\left[A_{\text{Pyr}(t,f_{\text{pyr}})}]\Phi_{\text{Rad}\left(t,f_{\text{rad}}\right)}(j\right)$ was divided for each bin value by the sum over all bins. The phase–amplitude distribution for each combination $\left(f_{\text{pyr}},f_{\text{rad}}\right)$ ($P\left(j,f_{\text{pyr}},f_{\text{rad}}\right)$) was obtained by the following equation, where *N* was the number of phase bins:

$$P\left(j,f_{\text{pyr}},f_{\text{rad}}\right)=\frac{\left[A_{\text{Pyr}\left(t,f_{\text{pyr}}\right)}]\Phi_{\text{Rad}\left(t,f_{\text{rad}}\right)}(j\right)}{\sum_{j=1}^{N}[A_{\text{Pyr}\left(t,f_{\text{pyr}}\right)}]\Phi_{\text{Rad}\left(t,f_{\text{rad}}\right)}(j)}$$

$$\;f_{\text{pyr}}=1,2,3\text{....}299,300\;\text{Hz},f_{\text{rad}}=1,2,3\text{...}20\;\text{Hz},$$

The joint entropy (*H*) of $\text{Rad}\left(t,f_{\text{rad}}\right)-\text{Pyr}\left(t,f_{\text{pyr}}\right)$ coupling was calculated as follows:

$$H\left(f_{\text{pyr}},f_{\text{rad}}\right)=-\overset{N}{\underset{j=1}{\sum }}P\left(j,f_{\text{pyr}},f_{\text{rad}}\right)\text{log}P\left(j,f_{\text{pyr}},f_{\text{rad}}\right).$$

When the joint distribution of $\Phi_{\text{Rad}\left(t,f_{\text{rad}}\right)}$ − $A_{\text{Pyr}(t,f_{\text{pyr}})}$ was uniform, the maximum value of joint entropy $H_{0}$ was obtained, as follows:

$$H_{0}=logN^{2}.$$

We then calculated the KL distance by subtracting the value *H*_0_ from *H*, and measured how much the $\Phi_{\text{Rad}\left(t,f_{\text{rad}}\right)}$ – $A_{\text{Pyr}(t,f_{\text{pyr}})}$ joint distribution deviated from the uniform joint distribution. Finally, MI was obtained by dividing the KL distance by the constant factor *H*_0_, which made the measure assume values between 0 and 1, as follows:

$$\text{MI}(f_{\text{pyr}},f_{\text{rad}})=\frac{H(f_{\text{pyr}},f_{\text{rad}})-H_{0}}{H_{0}}.$$

Thus, a larger MI value denoted a stronger coupling between the stratum radiatum and stratum pyramidale layers of the HC.

To investigate any statistical differences in coupling between the two groups, we calculated the *t* test of $\text{MI}(f_{\text{pyr}},f_{\text{rad}})$ for each combination of $f_{\text{pyr}}\;\text{and}\;f_{\text{rad}}$.

#### Spike sorting

Automated offline spike detection and sorting were performed on the wideband signals using the Klusta package (KlustaKwik; RRID:SCR_014480), developed for higher-density electrodes, and manually refined using the KlustaViewa (https://github.com/klusta-team/klustaviewa), or phy (https://github.com/kwikteam/phy) software package. The Klusta routines are designed to take advantage of the spatial arrangement of and spike timing from all probe channels simultaneously (32 channels for HC probe, 4 channels for MS tetrode) in constructing putative clusters (Rossant et al., 2016). Putative single units were required to have at least 100 spikes and a refractory period violation rate of <2% (interspike interval of <3 ms). For putative units isolated on the 32-channel HC probe, we further required a quality index of >0.95, a measure of cluster uniqueness relative to all other potential clusters in the dataset computed by the KlustaViewa software (Rossant et al., 2016). For putative units isolated on the MS tetrode, we additionally required the isolation distance (Harris et al., 2001) of each putative single unit to be ≥20 (Harris et al., 2003). The resulting curated putative single-unit clusters were then imported into MATLAB software, version R2017a, for visualization and further analysis using custom and built-in routines (MATLAB; RRID:SCR_001622). Wideband signals were bandpass filtered (0.6–6 kHz) for average waveform visualization only.

#### Subtype classification of putative single units in CA1

**Figure 4.**
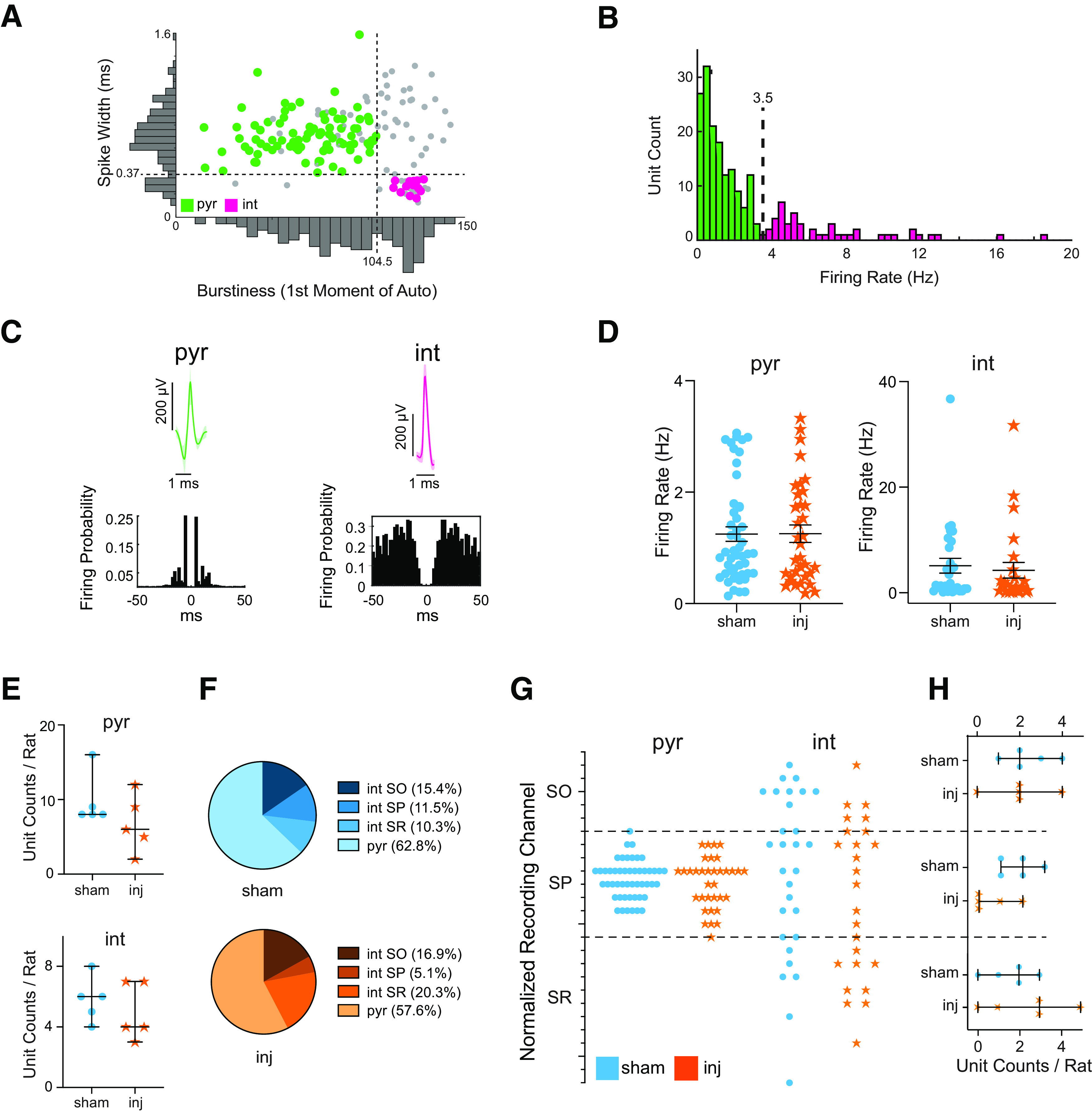
Neuronal firing rates and putative neuron number are preserved in CA1 following TBI. ***A***, ***B***, Division of isolated CA1 single units within SP into putative pyramidal cells and putative interneurons. ***A***, Dark gray bars indicate histograms of the first moment of the autocorrelogram (*x*-axis), with lower numbers indicating higher burstiness, and spike width (at 25% maximum height) of the unfiltered waveforms (*y*-axis). ***B***, Firing rate histogram of all isolated single units. Each feature reveals two distributions of single units, defining the following two clusters: magenta circles in the right bottom quadrant (narrow, tonic, fast spiking cells—putative interneurons); and green circles in the left top quadrant (wide, bursting, slower-spiking cells—putative pyramidal cells). Gray circles represent units that could not be subtyped. ***C***, Example of single isolated putative pyramidal cell (left) and putative interneuron (right). Top, Mean (±SD) waveforms. Bottom, Autocorrelograms expressed as firing probability as a function of time bin (bin size, 2 ms). Note in the putative pyramidal cell the wider waveform as well as the multiple short-latency peaks in the autocorrelogram that correspond to burst activity compared with the putative interneuron. ***D***, Overall mean (±SEM) firing rates of putative pyramidal cells (left) and interneurons (right) demonstrating no change in baseline activity between sham and injured animals. ***E***, No change in the median (with 95% CI) number of isolated putative pyramidal cells (top) or interneurons (bottom) per rat after injury. ***F***, Pie charts depict the relative distribution of putative neuronal subtypes across CA1 layers. ***G***, Laminar distribution of all isolated putative pyramidal cells (circles) and interneurons (stars) as a function of recording channel normalized to SP. Dotted lines indicate SO–SP and SP–SR borders. ***H***, Plots compare median (with 95% CI) number of isolated putative interneurons per rat between groups in each layer. There is no significant dropout of putative interneurons within any layer. Blue and orange represent sham and injured rats, respectively. Circles and stars represent putative pyramidal cells and interneurons, respectively. inj, Injured; pyr, pyramidals; int, interneurons.

We created a three-step process for classifying isolated HC putative single units on the 32-channel probe as either pyramidal cells or interneurons. In the first step, putative units were assigned to an anatomic layer (stratum oriens, stratum pyramidale, or stratum radiatum). We defined a five-channel span (100 μm) centered on the channel of maximum multiunit activity for each rat and defined this as stratum pyramidale. For each putative unit, if the channel of maximum amplitude fell within this band, it was assigned to stratum pyramidale. If the maximum amplitude was above this band, it was assigned to stratum oriens, and if below, it was assigned to stratum radiatum. If the maximum amplitude of a putative unit fell on a channel adjacent to the stratum pyramidale band, it was classified temporarily as a border unit. All nonborder putative units outside of stratum pyramidale were then classified, by definition, as interneurons, regardless of firing rate. In the second step, we modified the method of Csicsvari et al. (1999) of putative unit classification into pyramidal cells, or interneurons and applied it to all units assigned to stratum pyramidale or classified as border units. This approach computes the following three features of each putative unit: overall firing rate, a measure of averaged spike waveform width, and a measure of “burstiness” in the spike train of the unit. Spike width was calculated by extracting the waveform for each spike from the unfiltered wideband signal, which was then averaged and upsampled by a factor of 5. The peak amplitude of the averaged waveform was then detected, and its height was calculated by subtracting a baseline amplitude 1 ms before the peak. The spike width was then calculated as the time interval between the points along the averaged waveform that fell at 25% of the spike height, centered on the peak. Burstiness was defined as the first moment of the autocorrelogram, with smaller values indicating higher burstiness. The distributions of these three features across all putative stratum pyramidale units were plotted and inspected for natural clusters. Each feature revealed two clusters, and we thus made a simple division into two clusters for each feature using threshold values (see Fig. 4*B*). Putative pyramidal cells were defined as those units with a spike width above the determined threshold, a first moment of the autocorrelogram below the determined threshold (equating to high burstiness), and a firing rate below the determined threshold (division was at 3.5 Hz). Similarly, putative interneurons were defined as those units with a spike width below threshold, a first moment above threshold (equating to low burstiness), and a firing rate >3.5 Hz. In a final third step, those putative units classified as both interneurons and border cells were reassigned to stratum pyramidale if there were also isolated putative pyramidal cells with maximum amplitudes on the same channel, otherwise they were assigned either to stratum oriens or stratum radiatum, depending on which border they were located.

#### Subtype classification of putative single units in medial septum

To classify isolated single units in the MS as either putative GABAergic/glutamatergic (GABA/Glu) or putative cholinergic, we modified a method first described using intracellular and juxtacellular recordings in slice preparations (Matthews and Lee, 1991) and subsequently extended to *in vivo* recordings (King et al., 1998). This method relies solely on waveform shape by capturing an observed “inflection” of the repolarization phase of the action potential found to be characteristic of cholinergic neurons, but not GABAergic/glutamatergic neurons. Spectral power analysis of the waveforms from 1 to 16 kHz reveals increased power in higher-frequency bins in the “inflected” waveform group. We found that the distribution of the ratio of the fourth to second frequency bin revealed two natural clusters in both the sham and injured groups. The lower ratio, or “noninflected,” waveform cluster was then classified as putative GABAergic/glutamatergic cells. The higher ratio, or inflected, waveform cluster was then further refined by firing rate, as we expect cholinergic units to have firing rates generally <4 Hz. The firing rate distribution of the inflected subgroup revealed a low-firing cluster that was then classified as the putative cholinergic subgroup.

#### Entrainment of single units to local field potentials

For entrainment of putative HC or MS single units to the low-frequency oscillations, we bandpass filtered [8–20 or 2–5 Hz (“anesthesia theta”)] the LFP of the channel 425 μm below the center of stratum pyramidale, which corresponded to the channel of maximal power difference between groups within the 8–20 Hz range, and extracted the phase angles for each spike within each putative single unit using the Hilbert transform. Directional statistics were performed using MATLAB Circular Statistics Toolbox (CircStat; RRID:SCR_016651). For each putative unit, the mean resultant vector was calculated, giving the mean phase angle of entrainment for that unit, as well as the resultant vector length, a measure of spread of the individual phase angles about the mean. Putative units were then considered significantly entrained if the resultant vector length was greater than the threshold of nonuniform distribution calculated by the Rayleigh test (Berens, 2009). The mean angles of entrainment between significantly entrained sham and injured putative units as well as their distributions were compared using the two-sample Watson–Williams test and the two-sample Kuiper test, respectively.

For entrainment of putative HC single units to fast oscillations in stratum pyramidale we first extracted components of the stratum pyramidale LFP by wavelet transform (Morlet, 10 cycles) for each frequency (1–400 Hz, denoted as Pyr(t,f),f=1,2,3,...399,400 Hz). The phases for each frequency bin (denoted as $\Phi_{\text{Pyr}\left(t,f\right)},f=1,2,3,\text{...}399,400$) were obtained from the standard Hilbert transform. Next, the phases $\Phi_{\text{Pyr}\left(t,f\right)}$ were binned (18 bins, 20°), and the number of units per bin [*#*units(*j*)] was calculated. We denoted the normalized unit distribution over the phase bin *j* as follows:

$$P\left(j,f\right)=\frac{\#\text{units}(j)}{\sum_{j=1}^{N}\#\text{units}(j)}.$$

The joint entropy *H* was calculated as follows:

$$H\left(f\right)=-\overset{N}{\underset{j=1}{\sum }}P\left(j,f\right)\text{log}P\left(j,f\right).$$

We then calculated the MI for putative single unit–stratum pyramidale LFP entrainment, as per methods detailed above (see Phase–amplitude coupling section). A larger MI value denoted a stronger entrainment. As above, we used the Student’s *t* test to determine frequencies of significant differences in MI.

#### Spike-triggered averages

To investigate the influence of HC oscillatory activity on MS neuron firing patterns, we computed MS spike-triggered averages of the HC LFP recorded simultaneously with spiking activity in the MS. For each putative MS neuron, we averaged 1 s windows of the unfiltered HC LFP centered on each putative MS cell spike. Spike-triggered averages of putative units meeting criteria for “CA1 modulated” (see below) were then averaged within the sham and injured groups. To quantify differences in the averaged spike-triggered averages between the sham and injured groups, we calculated the absolute difference between the maximum positive and maximum negative voltage deflections in the averaged tracings for each unit, making between-group comparisons using Welch’s *t* test.

#### Peristimulus time histograms

For each putative CA1-modulated MS unit the concurrently recorded HC LFP within stratum radiatum was bandpass filtered from 8 to 20 Hz or from 2 to 5 Hz (anesthesia theta). The troughs of the resulting oscillation were detected, and the times of these events were used as a trigger for the creation of the peristimulus time histogram (PSTH; 100 ms window; bin size, 2 ms) for each putative CA1-modulated MS unit. Because individual MS units had different overall firing rates for each bin, the number of spikes was divided by the total number of spikes for that putative unit to derive a firing probability that could be compared across units.

#### Data availability

Custom MATLAB scripts written for electrophysiology data analysis are described above. Custom scripts and data presented in this article are available on request.

## Results

### Injury physiology and behavioral deficits confirm moderate to severe TBI

The median delivered pressure wave during lateral FPI in the injured group (*N* = 11) was 2.30 atm (range, 2.01–2.46 atm), and the median apnea time after injury was 27 s (range, 0–105 s), consistent with a moderate to severe level of injury (Smith et al., 1991). One animal had a 5 s seizure in the immediate postinjury period. One animal experienced a large subdural hematoma after FPI and was excluded. No dural breaches occurred.

Both the injured group (*N* = 10) and sham group (*N* = 10) underwent preinjury training and postinjury testing (postinjury day 1) on the Morris water maze. The median calculated memory score for recall of the hidden platform during postinjury testing was significantly lower in the injured group compared with the sham group [injured group: 21.21 (range, 0–86.35); sham group: 65.84 (range, 4.67–113.9); Mann–Whitney test, *p* = 0.012], while there was no difference in swimming speed (Mann–Whitney test, *p* = 0.74). These hippocampus-relevant performance deficits are also consistent with a moderate to severe injury (Smith et al., 1991).

### Hippocampal power losses after FPI are anatomic layer and frequency band specific

**Figure 1.**
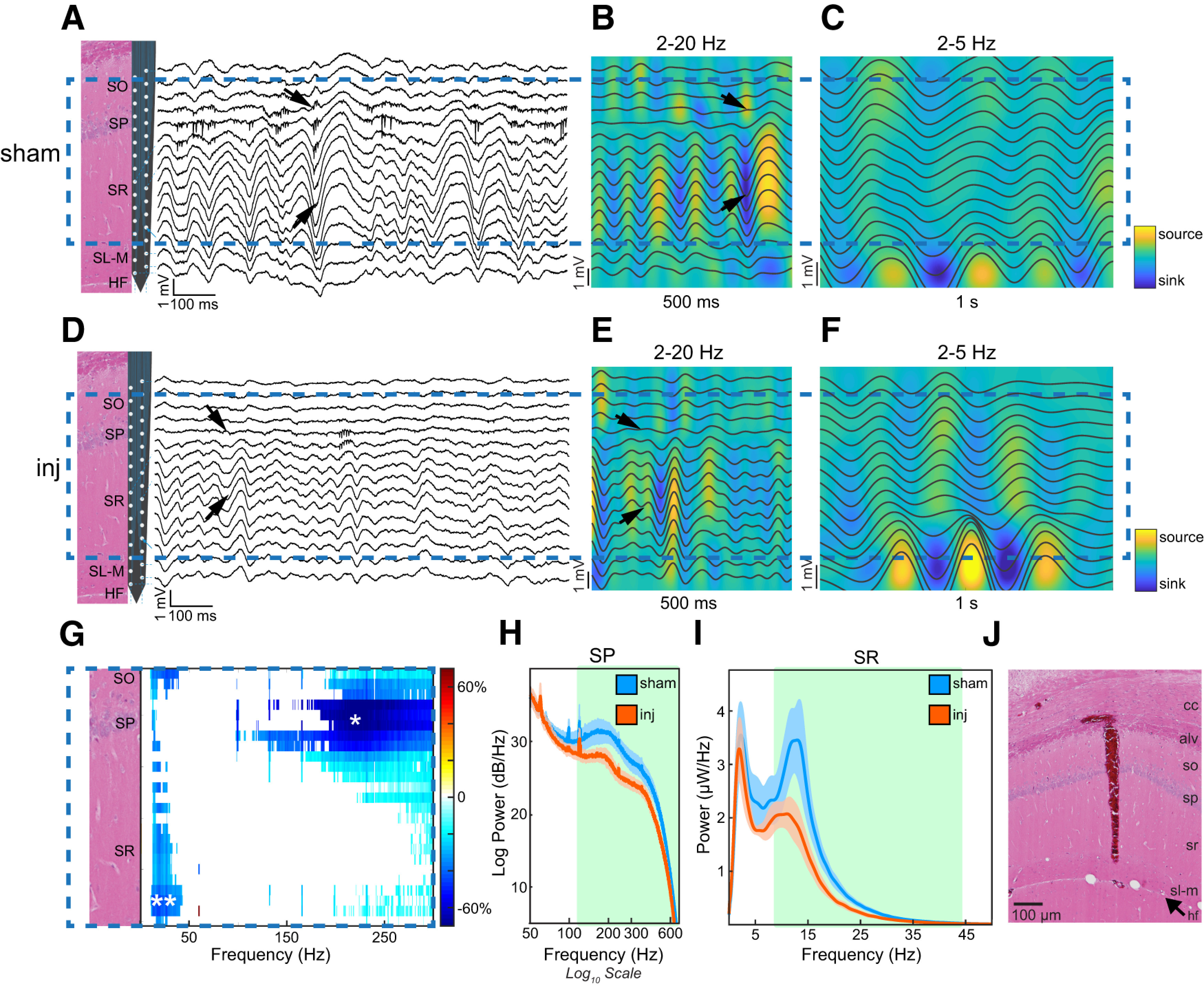
Laminar power profile in CA1 following TBI demonstrates attenuation of rhythmic input into stratum radiatum and reduction of high-frequency oscillations within stratum pyramidale, but preservation of anesthesia theta. ***A***, ***D***, One second example trace of wideband LFP across CA1 laminar structure in a single sham rat (***A***) and a single injured rat (***D***). H&E-stained section of dorsal CA1 and drawing of a 32-channel silicon probe illustrate the approximate positions of channels relative to CA1 layers. A dominant ∼10–15 Hz oscillation in the sham is noticeably attenuated in the injured case. Arrows indicate phase reversal across SP, consistent with sharp-wave input from CA3. ***B***, ***C***, ***E***, ***F***, Current source density analysis of a single 500 ms and a separate 1 s LFP segment, illustrating epochs of prominent 10–15 Hz SR oscillations, or 2–5 Hz anesthesia theta oscillations, respectively, from one sham rat (***B***, ***C***) and one injured rat (***E***, ***F***), filtered from 2 to 20 Hz (500 ms segment; ***B***, ***E***), or from 2 to 5 Hz (***C***, ***F***). Blue indicates a sink, while yellow indicates a source. Dark gray lines show filtered LFP. Arrows indicate rhythmic sink in SR with accompanying source in SO. Note maximal CSD sink within SR and sharp phase reversal across SP in the ∼10–15 Hz oscillation, consistent with sharp wave-like inputs, while maximal CSD sink occurs near SL-M and phase shift is gradual along the dendritic arbor from SO to SL-M in the 2–5 Hz oscillation, consistent with a theta laminar pattern. Dotted blue box indicates channels common to all animals after alignment to SP, incorporating portions of SO, SP, and SR. ***G***, Masked heat map of significant differences (Student’s *t* test, *p* < 0.05) in power between the sham and injured groups across 25 channels aligned to SP. Colored pixels represent a significant percentage change in power from sham to injured groups. White indicates no significant difference. A single white asterisk marks the SP channel at which the power spectrum in ***F*** is calculated. Double white asterisks mark the SR channel at which the power spectrum in ***G*** is calculated. ***H***, ***I***, Averaged power spectral densities from 50 to 700 Hz in SP (***H***) and from 2 to 50 Hz in SR (***I***). Power density is expressed as log power density (dB/Hz), and frequencies are displayed on a logarithmic scale in ***H*** for better visualization. Blue lines indicate sham, orange lines indicate injured. Error bars represent the SEM. While no difference is present in the 2–5 Hz peak, there is a significant loss of power in the 10–15 Hz peak and >120 Hz after injury. Green background indicates frequency ranges of significant differences in power between groups in ***H*** (121–700 Hz) and ***I*** (10.5–43 Hz; Student’s *t* test, *p* < 0.05). ***J***, Representative H&E-stained section of the dorsal hippocampus CA1 demonstrating a probe artifact with tip sitting just above SL-M. HF, hippocampal fissure.

Five animals each from the sham and injured groups underwent acute recordings from the hippocampus under isoflurane anesthesia [median isoflurane levels: sham group, 1.63 (range, 1.5–2); injured group, 1.5 (range, 1.5–1.75); Mann–Whitney test, *p* = 0.76] 7 d after FPI or sham injury. A 32-channel silicon probe (Poly-2 design) capable of recording LFP and single-unit activity was inserted into the dorsal CA1 region of the ipsilateral hippocampus, spanning multiple anatomic layers, including stratum oriens (SO), stratum pyramidale (SP), stratum radiatum (SR), and a portion of stratum lacunosum-moleculare (SL-M) (Fig. 1*A*,*D*,*J*).

We first examined whether there were LFP power differences between the sham and injured groups that were frequency specific and anatomic layer specific. To quantify this, we calculated the power spectral density at each anatomically distributed recording channel from 1 to 300 Hz for each animal. Channels were then spatially aligned to the channel of peak spiking activity within SP (see Materials and Methods), giving a total of 25 channels of overlap extending from SO to the bottom of SR. We compared the power density between the sham and injured groups at each channel and frequency, generating a map of significant differences (repeated-measures ANOVA, *p* < 0.05). Although there were no regions of increased overall oscillatory power after injury, the power was significantly reduced within SR, with peak reduction of 46.7% at a frequency of 13.5 Hz (range, 10.5–43 Hz) at its deep extent, and within SO with peak reduction of 45% at a frequency of 26 Hz (range, 10–39 Hz; Fig. 1*G*,*I*).

To better characterize this ∼10–15 Hz SR/SO oscillation, we visually inspected the unfiltered LFP in sham and injured rats. The sham LFP revealed a prominent oscillation in the ∼10–15 Hz range [median frequency of peak power: 12 Hz (range, 10–15 Hz)] detectable at all recording sites spanning the CA1 anatomic layers, but with greatest amplitude in SR (Fig. 1*A*). This oscillation was present with a 180° phase reversal within SO. To further refine the anatomic specificity of this oscillation, the CSD was calculated for representative segments of the LFP exhibiting this oscillation, revealing a prominent sink centered in SR and an associated source in SP (Fig. 1*B*). Inspection of the LFP and associated CSD in the injured animals revealed a similar oscillation but with an apparent lower amplitude (Fig. 1*D*,*E*). The median frequency of peak power in the injured group was 9.5 Hz (range, 9–12 Hz; Mann–Whitney test, *p* = 0.09 vs sham). Together, these data suggest that there is a prominent coordinated synaptic input into SR in both the sham and injured groups, but that the strength of this input is significantly reduced after injury, either because of reduced presynaptic input, reduced coordination of that input, or both.

Although present equally in sham and injured animals, a second prominent power peak was observed in SR within the ∼2–5 Hz range [median frequency, 2 Hz (sham and injured groups); Mann–Whitney test, *p* = 0.64; Fig. 1*I*]. While hippocampal theta oscillations in the awake, exploring rat are in the 4–10 Hz range, it has been reported that oscillations with a similar laminar pattern within rodent CA1 can be observed under isoflurane anesthesia in the 2–5 Hz range (Lustig et al., 2016), here termed anesthesia theta. To further explore whether these ∼2–5 Hz oscillations were likely to represent anesthesia theta oscillations (analogous to awake theta oscillations), we filtered the wideband LFP from 2 to 5 Hz and located epochs of increased power within this band for inspection of the laminar profile. A representative 1 s epoch of LFP and CSD illustrating prominent anesthesia theta from the sham and injured groups are depicted in Figure 1, *C* and *F*. These oscillations demonstrate the largest amplitude coupled with the maximal CSD sink within SL-M, suggesting that significant coordinated synaptic input into SL-M is present (Fig. 1*C*,*F*). There is also a progressive phase shift of this oscillation from SO to SL-M. These features are characteristic of 4–10 Hz theta oscillations during the awake state (Buzsáki, 2002) and also observed in the 2–5 Hz range under isoflurane anesthesia (Lustig et al., 2016). As peak power of theta oscillations occurs in SL-M (Buzsáki, 2002), it is possible we failed to capture a difference in theta power in the aligned channel data, as the deepest channel was still located within SR. We therefore compared the spectral power between subgroups of the sham (*n* = 3) and injured (*n* = 4) cohorts that had recording probes at least 100 μm below the deepest aligned, common channel, capturing SL-M, finding no differences in power from 2 to 5 Hz. These data suggest that under the present levels of isoflurane anesthesia, no differences in anesthesia theta power can be observed between sham and injured animals.

**Figure 3.**
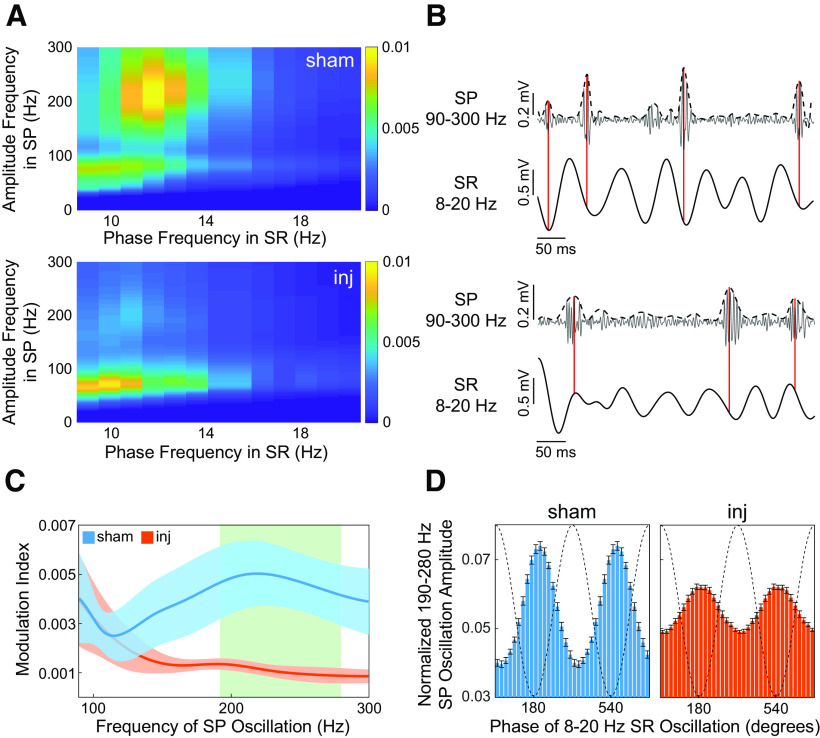
TBI reduces phase-amplitude coupling between rhythmic stratum radiatum input and high-frequency oscillations in stratum pyramidale. ***A***, Mean sham (top) and injured (bottom) comodulograms depicting the modulation index of phase frequency (8–20 Hz) in SR with amplitude frequency (0–300 Hz) in SP. Warmer colors indicate higher modulation indices. Note the strong coupling between the ∼10–15 Hz phase in SR and high-frequency oscillation (∼200–300 Hz) amplitude in the sham group, which is lost after injury. ***B***, A 500 ms CA1 tracing from a single sham rat (top) and an injured rat (bottom), demonstrating phase–amplitude coupling between dominant SR rhythmic input and high-frequency oscillations in SP. Top tracing is the normalized bandpass filtered LFP (90–300 Hz) in SP. The dotted line is a smoothed envelope for visual clarity. Bottom tracing is the simultaneous bandpass filtered LFP (8–20 Hz) in SR. Vertical red lines highlight alignment of high-frequency oscillatory peaks to a consistent phase of the low-frequency input into SR in the sham group. This alignment becomes inconsistent in the injured group. ***C***, Mean (±SEM) modulation index of the 8–20 Hz SR input as a function of high-frequency oscillation amplitude in SP. Green background indicates a high-frequency band (190–280 Hz) of significant loss of modulation by the SR input after injury. ***D***, Mean (±SEM) phase–amplitude coupling between the 8 and 20 Hz phase in SR and the 190–280 Hz amplitude in SP. Note the maximum high-frequency amplitude in SP just follows the minimum low-frequency phase, which corresponds to the phase of maximum inward current in SR. Dotted lines represent the phase of the 8–20 Hz oscillation. Blue and orange represent the sham and injured groups, respectively, in ***C*** and ***D***.

While significant power differences within low frequencies were observed within SO and SR, layers containing pyramidal cell dendritic arbors, a wide band of high-frequency power loss was observed principally within the pyramidal cell layer itself (SP; Fig. 1*G*). Power peaks within the ∼100–250 Hz band in the sham group and a weaker peak in the injured group were observed within SP (Fig. 1*H*). Inspection of the filtered SP LFP (90–300 Hz) reveals brief (∼50–100 ms) oscillatory events of increased amplitude in both groups (see Fig. 3*B*). However, we found a reduction in power after injury across a wide frequency range of 121–700 Hz, with peak reduction of 74.4% power at a frequency of 231.5 Hz in SP (Fig. 1*G*,*H*). High-frequency oscillations recorded within SP likely reflect local processes, whether they are coordinated spiking activity of local neurons or synaptic activity between pyramidal cells and local interneuronal networks (as occurs in ripple events; see Discussion; Schomburg et al., 2012). High-frequency power losses after injury may indicate a loss of overall local neuron firing (whether because of reduced firing rates or reduced neuron numbers), an impairment in coordination among the firing patterns of local neurons, or a loss of pyramidal cell–interneuron interactions leading, for example, to reduced ripple events (brief oscillations observed in SP within the ∼150–250 Hz band; Schomburg et al., 2012; Buzsáki, 2015). Together the power losses we observe in ipsilateral CA1 7 d after TBI are frequency and anatomic layer specific, reflecting both a reduction in coordinated synaptic input into SR and a loss or discoordination of local spiking and/or synaptic activity within SP.

### Histopathology confirms injury severity and reveals widespread pathology of major hippocampal afferents, supporting loss of synaptic input into stratum radiatum after FPI

A separate subset of animals (sham vs injured and without electrophysiological testing) were sacrificed at 7 d post-FPI for histopathological analysis to characterize the pattern and distribution of pathology within the model.

**Figure 2.**
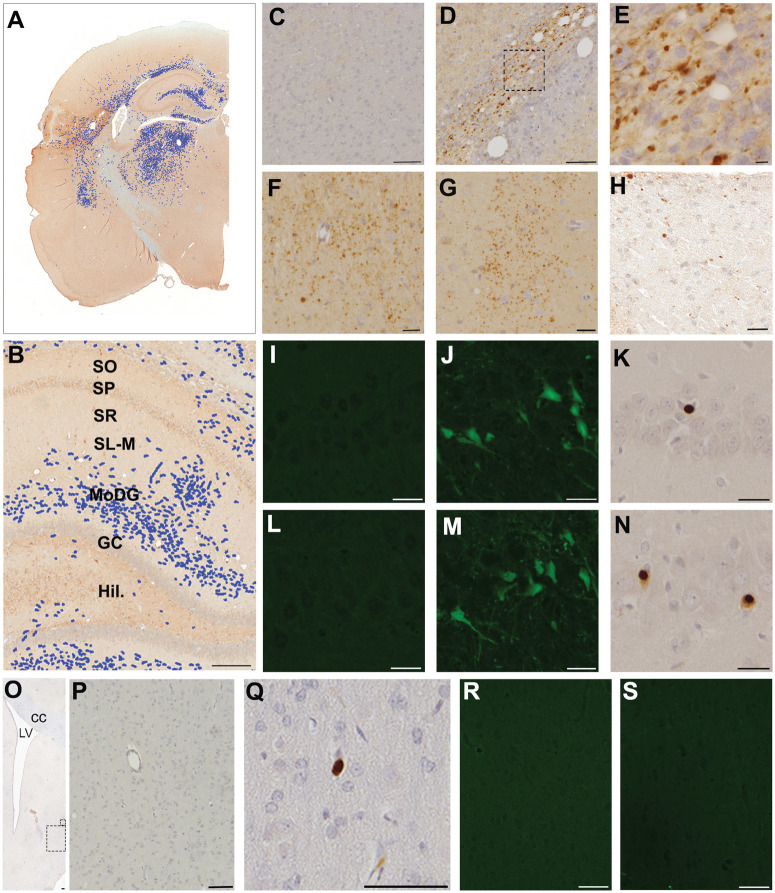
Neuronal and axonal injury of intrahippocampal circuitry, hippocampal afferent input pathways, and medial septum. ***A***, Whole-hemisphere map of APP-positive axonal pathology generated via manual annotation of digitally scanned APP-stained tissue section using ImageScope software (Leica Biosystems). Swollen axonal profiles were tagged (blue) to provide representative distribution map. ***B***, Blow up of the hippocampal region identified in ***A***, illustrating APP-positive axonal pathology within SR (target of Schaffer collaterals), as well as SL-M and MoDG, targets of the angular bundle (temporoammonic and perforant pathways, respectively). ***C***, Sham animal without any APP-positive axonal pathology. ***D–H***, Extensive axonal pathology as evidenced by pathologic APP accumulation in axonal varicosities and disconnected terminal bulbs in the angular bundle (***D***), high-magnification image of region identified in ***D*** (***E***), stratum radiatum (***F–G***), and fimbria/fornix (***H***) at 7 d post-FPI. ***I***–***K***, Representative example of a sham animal (***I***) without any Fluoro-Jade C-positive cells in the CA1 subregion of the hippocampus. In contrast, numerous Fluoro-Jade C-positive (***J***) and Ab246-positive (***K***) cells indicate neuronal degeneration in CA1 of hippocampus at 7 d post-FPI. ***L–N***, Similarly, an absence of Fluoro-Jade C-positive cells in the CA3 subregion of the hippocampus in a sham animal (***L***) versus multiple Fluoro-Jade C-positive (***M***) and Ab246-positive (***N***) cells in CA3 at 7 d post-FPI. ***O–Q***, Representative Ab246 staining in the medial septum at 7 d post-FPI. ***P***, High-magnification image of the region shown by the large box in ***O***, displaying an absence of Ab246 immunoreactivity. ***Q***, In the same case shown in ***O*** and ***P***, a single Ab246 cell was observed in the medial septum (high-magnification image of the small box shown in ***O***). Note this was the only Ab246-positive cell observed in the medial septum across all injured animals. ***R***, Representative image showing an absence of Fluoro-Jade C staining in the medial septum of a sham animal. ***S***, A similar absence of Fluoro-Jade C staining in the medial septum in the same injured animal/region as shown in ***O*** and ***P***. Scale bars: ***B***, ***O***, ***P***, 100 μm; ***C***, 10 μm; ***D–H***, ***I–N***, 25 μm; ***Q–S***,  20 μm. MoDG, Molecular layer of dentate gyrus; GC, granule cell layer of dentate gyrus, Hil, hilus.

H&E staining revealed a focal intraparenchymal lesion consistent with previous descriptions of the model (Pierce et al., 1998). Notably, the lesion was not directly below the impact site, but rather was observed laterally, affecting the temporoparietal cortex and adjacent corpus callosum. In addition to the focal lesion, regionally more diffuse injury could be observed, as evidenced by neuronal pyknosis in the hippocampus and underlying thalamus. IHC revealed axonal pathology morphologically consistent with previous descriptions in both human TBI and experimental TBI models (Gentleman et al., 1993; Johnson et al., 2013a,b, 2016; Weber et al., 2019). Specifically, abnormal APP accumulations were observed within tortuous varicose profiles and disconnected or terminal axonal bulbs secondary to transport interruption (Fig. 2*D–H*). In addition to the site of the focal lesion as described above, axonal pathology was observed to be more widespread throughout the brain white matter, including extensive pathology in the corpus callosum, ipsilateral thalamus, hippocampus, as well as minimal pathology in the contralateral hemisphere (Fig. 2*A*,*B*). As such, the model as described recapitulates both focal and diffuse pathologies, including cortical contusion and widespread axonal pathology, commonly observed following moderate to severe TBI in humans. In addition, the extent and distribution of pathology appears comparable to a degree of injury in the moderate to severe range for FPI (Smith et al., 1997).

Given the electrophysiological evidence of reduced coordinated synaptic input into CA1 SR, we asked whether there was structural evidence for impaired afferent projections into CA1 SR within the model. APP-positive axonal pathology was observed in all injured (*n* = 4) animals within SR and was most prominent medially (Fig. 2*A*,*B*,*F*,*G*).

However, axonal injury was not limited to SR, and APP-positive axonal swellings were consistently observed in all injured animals within the angular bundle (Fig. 2*D*,*E*) and the fimbria fornix (Fig. 2*H*), as well as within the SL-M of CA1 and the molecular layer of the dentate gyrus, the layers of termination of the angular bundle (via the temporoammonic and perforant pathways, respectively; Fig. 2*B*). Axonal pathology was minimally observed in stratum oriens, again more prominent closer to the midline.

Evidence of ongoing cell death was also observed within the CA3 subfield, a major source of excitatory projections into SR of the CA1 subfield. Specifically, in all four injured animals Fluro-Jade C-positive neurons were observed in CA3, with three of four animals also displaying immunoreactivity for Ab246 (apoptosis) in a subset of cells (Fig. 2*M*,*N*). While there was also evidence of cell death within CA1 in all injured animals (see Fig. 2*J*,*K*), this was maximal at the junction between CA1 and CA2, but absent more medially within CA1.

**Figure 6.**
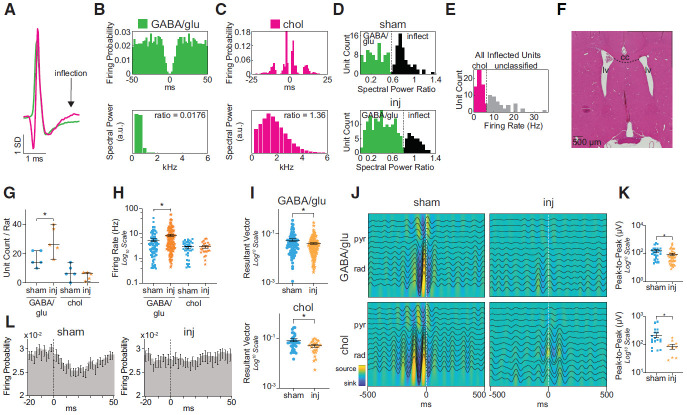
TBI leads to a reduction in rhythmic CA1 inhibition of medial septum. ***A***, Unfiltered averaged waveforms of isolated MS sham putative GABAergic/glutamatergic (magenta) and putative cholinergic (green) single units, amplitude normalized to highlight waveform shape differences. Note “inflection” during repolarization, characteristic of cholinergic neurons. ***B***, ***C***, Top, Autocorrelograms of same single MS units. Bottom, Power spectrum (1–16 kHz, 25 bins) of same single MS units. Spectral power ratio is of fourth to second frequency bins. Putative cholinergic waveform inflection manifests as increased power at higher frequencies. ***D***, Histograms of spectral power ratios for all sham (top) and injured (bottom) MS units. Two distributions are apparent, defining putative GABAergic/glutamatergic and cholinergic populations. ***E***, Firing rate histogram of all putative single MS units meeting spectral ratio criteria as “inflected” units, revealing a distinct subpopulation of low-firing units, which we have classified as putative cholinergic units. ***F***, Representative H&E-stained section of the medial septum with tetrode artifact. ***G***, Increased number of median (with 95% CI) isolated putative GABAergic/glutamatergic units per rat after injury, but no change in putative number of cholinergic units. ***H***, Increased mean firing rate of isolated putative GABAergic/glutamatergic units after injury, but no change in putative cholinergic unit firing rates, suggesting increased excitation or loss of inhibition. ***I–K***, Modulation of isolated MS units by 8–20 Hz oscillation in SR of CA1. ***I***, Reduction in the mean strength of MS unit modulation (resultant vector) by CA1 8–20 Hz in putative GABAergic/glutamatergic (top) and cholinergic (bottom) subgroups. ***J***, MS unit spike-triggered average of CA1 8–20 Hz filtered LFP, averaged across all putative GABAergic/glutamatergic (top) and cholinergic (bottom) MS units significantly modulated by the CA1 oscillation in the sham (left) and injured (right) groups. Injured color maps normalized to sham color maps. Note reduction in ∼10–15 Hz CA1 SR oscillation just preceding the MS action potential (dotted white line). ***K***, Reduction in the peak positive to the peak negative voltage difference in the averaged spike-triggered averages of the deepest channel in stratum radiatum depicted in ***J*** for both putative GABAergic/glutamatergic and cholinergic MS units. ***L***, Averaged peristimulus time histograms for all putative MS units (GABAergic/glutamatergic and cholinergic) significantly modulated by CA1 8–20 Hz SR oscillation, triggered off the trough of that oscillation (dotted lines) in the sham (left) and injured (right) groups. As the maximal unit firing probability in CA1 occurs near the 8–20 Hz filtered SR trough (Fig. 5), the lowest MS unit firing probability occurring just after this trough in the sham group, but not in the injured group, suggests that coordinated rhythmic inhibition of MS by CA1 is diminished after injury. Single asterisks indicate a significant difference (*p* < 0.05).

**Figure 5.**
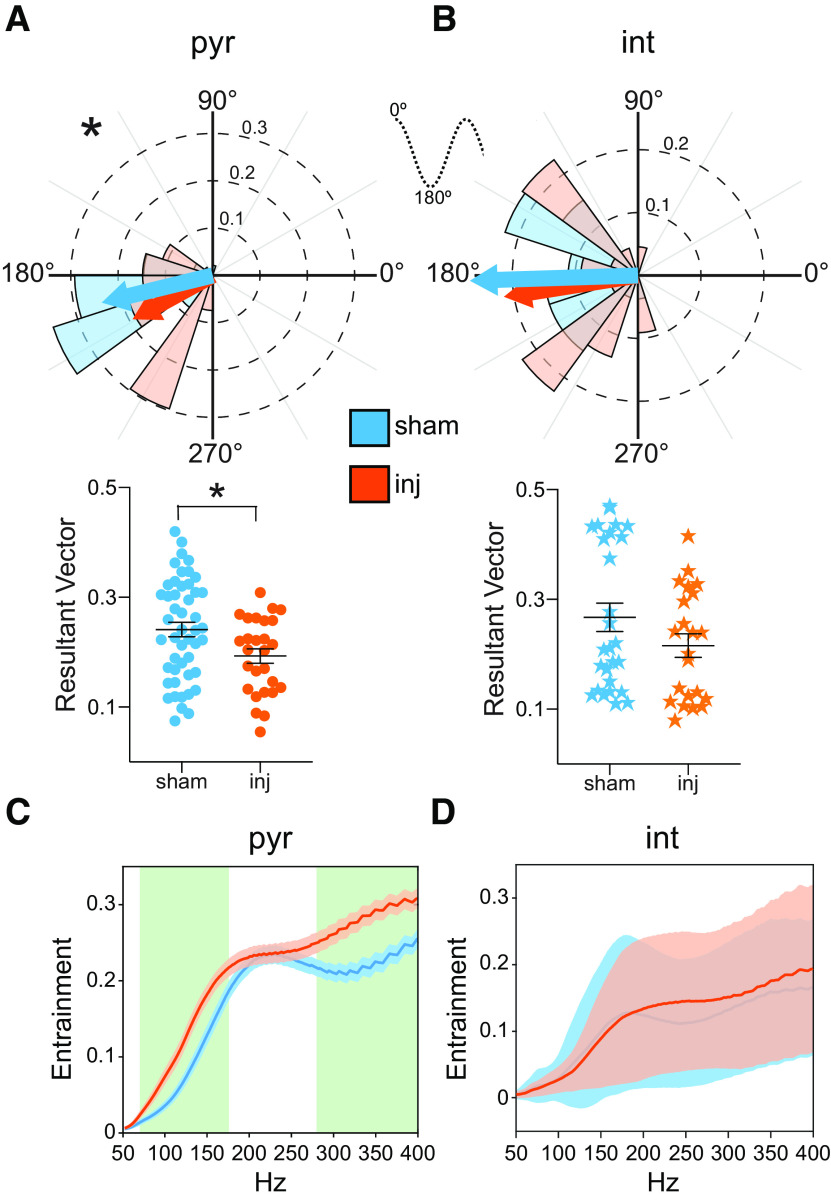
Despite normal firing rates, CA1 neuronal entrainment to rhythmic stratum radiatum input and local high-frequency oscillations is altered following injury. ***A***, ***B***, Entrainment of single neurons to rhythmic SR oscillation (8–20 Hz). Top, Polar histogram depicting preferred phase of entrainment of putative CA1 pyramidal cells (***A***) or interneurons (***B***) that are significantly entrained to 8–20 Hz oscillation in SR. There is a significantly broader distribution of preferred phase angles among the injured pyramidal cells as well as a subset that appears to entrain with a ∼30° offset. Solid arrow vectors indicate the mean phase angle of entrainment with length equal to the mean resultant vector. Asterisk indicates significant difference in distribution (*p* < 0.05). Dotted sinusoidal wave illustrates phase angle of LFP signal. Bottom, Comparison of the group mean (±SEM) resultant vector lengths for each entrained putative pyramidal cell (***A***) or interneuron (***B***). Resultant vector indicates the variance of individual spike phases about the mean for a given single putative neuron (resultant vector of 1 indicates identical phases for all spikes), giving a measure of the degree of entrainment of a given putative neuron. The degree of entrainment of the pyramidal neurons was significantly lower following injury, suggesting a decrease in the ability of these neurons to follow the oscillation. ***C***, ***D***, Degree of entrainment (mean ± SEM) of significantly entrained putative pyramidal neurons (***C***) and interneurons (***D***) to local high-frequency oscillations (50–400 Hz) as a function of frequency. A focal peak frequency band (∼200–250 Hz) in sham putative pyramidal cell entrainment becomes a broader frequency range of increased entrainment after injury, incorporating higher and lower frequencies. Green background indicates frequencies of significant difference between groups (*p* < 0.05). Blue and orange represent sham and injured, respectively.

Together, these data suggest that FPI can result in pathologic change that may account for impaired afferent input into SR after injury. Specifically, histopathological characterization of the model suggests that this altered input may be coming from CA3 via Schaffer collaterals, whether because of CA3 pyramidal cell loss and consequently reduced CA1 presynaptic activity or because of a loss of coordination of that input.

Within the medial septum there was no evidence of any axonal pathology or Fluoro-Jade C-positive cells in any injured animals (Fig. 2*S*). Ab246 immunoreactivity was also absent in three injured animals, with the remaining injured animal displaying just a single positive cell (Fig. 2*O*–*Q*). There was similarly no evidence of overt cell death within stratum oriens.

Notably, no axonal pathology or evidence of cell death was observed in any sham animals in any regions (Fig. 2*C*,*I*,*L*,*R*).

### FPI reduces coupling of local CA1 stratum pyramidale high-frequency activity with stratum radiatum synaptic input

The observed post-FPI power loss of a 10–15 Hz oscillation in SR could reflect loss of coordinated CA3 input, as CA3 Schaffer collaterals primarily terminate in SR. Since CA3 sharp-wave input drives the occurrence of high-frequency oscillations such as ripples in CA1 SP in the nonanesthetized rat, we hypothesized that our observed reduction in SR input would exert less influence over local CA1 activity in SP, manifesting as a loss of temporal coupling between the low-frequency SR input and high-frequency oscillations in SP in addition to the observed high-frequency power loss in SP. Using the spatially aligned data across all animals, we calculated the coupling of the oscillation phase over time in SR across frequencies from 8 to 20 Hz and the oscillation amplitude over time in SP from 1 to 300 Hz. The strength of this coupling is quantified by the MI in Figure 3*A*. Oscillatory power in the high-frequency range occurred in bursts lasting ∼50 ms (Fig. 3*B*). We found strong specific phase–amplitude coupling between the ∼11 and 13 Hz phase in SR and the ∼150–300 Hz amplitude in SP in the sham group, which appeared to be severely attenuated in the injured group (Fig. 3*A*). In contrast, a coupling between the SR phase and amplitude of gamma-range oscillations (30–80 Hz; Jia and Kohn, 2011) in SP appears present in both groups. Given that the ∼11–13  and ∼150–300 Hz frequency ranges fell within the frequencies of observed power drops after FPI, we directly compared the MIs between groups with a phase frequency range of 8–20 Hz and an amplitude frequency range of 90–300 Hz (Fig. 3*C*). We found that FPI produced a significant loss in modulation of the high-frequency amplitude in SP (190–280 Hz) by the low-frequency phase in SR (11–13 Hz; Student’s *t* test, *p* < 0.05; Fig. 3*D*). Further, the maximum amplitude in the high-frequency signal aligned closely with the phase of maximal downward deflection in the low-frequency signal, which corresponds to the phase of maximal current influx into SR (Fig. 3*D*)—a relationship expected if the low-frequency signal reflects SR synaptic input driving local neuronal activity in SP (Buzsáki, 2015). In contrast, the coupling between SR phase and SP mid-gamma oscillatory amplitude was not statistically different (Student’s *t* test, *p* > 0.05). These results suggest that local neuronal activity in SP (observed as high-frequency oscillations) has been partially decoupled from the observed oscillatory input into SR after FPI. This may be due directly to loss of that input, disrupted coordination among the afferent neurons, or a reduced ability of local CA1 neurons/networks to properly respond to that input.

### Local CA1 neurons maintain normal firing rate capacity after FPI

One possible explanation of the observed high-frequency power losses within SP after FPI could be reduced CA1 neuron firing rates, reflecting an impaired ability to achieve a normal response to synaptic input. To address this possibility, we explored whether the firing properties of isolated CA1 neurons were altered after injury. We isolated single units from the raw signal and then classified them into putative pyramidal cells and putative interneurons (Fig. 4*B*). Using this scheme, we were able to classify 78 of 116 (67%) sham units and 59 of 86 (69%) injured units. We isolated a total of 49 putative pyramidal cells and 34 putative interneurons in the sham group and 34 putative pyramidal cells and 25 putative interneurons in the injured group. We found no significant difference in the median number of putative pyramidal cells per rat [sham group, 8 (range, 8–16); vs injured group, 6 (range, 2–12); Mann–Whitney test, *p* = 0.33; Fig. 4*D*, top], nor in the median number of putative interneurons per rat in the injured group [sham group, 6 (range, 4–8); vs injured group, 4 (range, 3–7); Mann–Whitney test, *p* = 0.58; Fig. 4*D*, bottom]. We further found no differences in putative interneuron numbers within each anatomic layer (SO, *p* = 0.80; SP, *p* = 0.12; SR, *p* = 0.51; Fig. 4*G*). Strikingly, the mean firing rates were also unchanged after injury for putative pyramidal cells (sham group, 1.25 ± 0.91 Hz; vs injured group, 1.26 ± 0.91 Hz; Welch’s *t* test, *p* = 0.98), putative interneurons (sham group, 5.12 ± 7.50 Hz; vs injured group, 4.26 ± 7.51 Hz; Welch’s *t* test, *p* = 0.68), and putative interneurons within each layer separately (SO, *p* = 0.09; SP, *p* = 0.89; SR, *p* = 0.13), demonstrating an overall preservation of firing rates between sham and injured animals (Fig. 4*D*).

### Injured neurons lose proper entrainment to rhythmic stratum radiatum input and local high-frequency oscillations

An important presumed function of oscillatory synaptic input in the hippocampus is to precisely organize the firing patterns of local neurons, leading to neurons firing at a particular phase relative to an oscillation or shifting that phase in a predictable fashion over time for encoding purposes (O’Keefe and Recce, 1993). Although we found that overall neuron firing rates remained stable among isolated single units after FPI, it is possible that they are unable to appropriately phase lock to organizing oscillatory activity. To directly address whether CA1 neuron firing patterns are influenced by the observed oscillatory SR input and assess whether this influence is impaired after injury, we investigated the phase relationship of spiking patterns of isolated single neurons in CA1 to the observed ∼10–15 Hz oscillatory input in SR (so-called “entrainment”; Fig. 5); 48 of 49 (98%) putative pyramidal cells were significantly entrained in the sham group, while only 27 of 34 (79%) cells were entrained in the injured group, a drop in number of nearly 20% (χ^2^ test, *p* < 0.0001). In contrast, the number of putative interneurons entrained was similar in sham and injured animals: 27 of 29 (93%) putative interneurons in the sham group, and 22 of 25 (88%) in the injured group (χ^2^ test, *p* = 0.31).

To assess the strength of entrainment of individual neurons to this oscillation, we used the resultant vector, which is a measure of how tightly distributed the phases of each action potential in the spike train of a given neuron are around the mean phase of all action potentials (a resultant vector of 1 indicates that all spike phases are identical and the neuron is strongly entrained, while a value of 0 indicates that there is no preferred phase and no entrainment). We found a significant drop in the mean resultant vector, or strength of entrainment, of putative pyramidal cells (sham group, 0.24 ± 0.09; injured group, 0.19 ± 0.07; Welch’s *t* test, *p* = 0.012), but not interneurons (sham group, 0.27 ± 0.13; injured group, 0.22 ± 0.10; Welch’s *t* test, *p* = 0.13) after injury (Fig. 5*A*,*B*).

We then asked how well neurons as a group were entraining to the proper phase after injury. Surprisingly, putative CA1 pyramidal cells in the injured group were unable to maintain a consistent phase preference relative to the SR oscillatory input compared with those in the sham group, displaying both a significantly wider variability in their preferred phase of entrainment (Kuiper test, *p* < 0.05; Fig. 5*A*, top), as well as an altered mean phase of entrainment (sham group, 193.8 ± 24.9°; injured group, 208.7 ± 32.7°; Watson–Williams test, *p* = 0.039; Fig. 5*A*, top). Visual inspection of the circular distributions in Figure 5*A* strongly suggests a secondary preferred phase of ∼240° in the injured group, but not in the sham group. There were no differences in the interneuron mean preferred phase (sham group, 181.8 ± 35.6°; injured group, 189.0 ± 50.0°; Watson–Williams test, *p* = 0.61) or in the wide distribution of preferred phases between groups (Kuiper test, *p* = 0.13; Fig. 5*B*, top).

We also examined CA1 neuron entrainment to SP high-frequency oscillations, revealing that it had become less focused in the pyramidal cells (Fig. 5*C*). In sharp contrast to SR oscillation entrainment deficits after injury, there were significant increases in the entrainment of putative pyramidal cells to the 55–175 and 280–400 Hz oscillatory activity in SP (Student’s *t* test, *p* < 0.05; Fig. 5*C*), broadening the peak of entrainment across a wider range of high-frequency oscillations after injury. Interneuron entrainment in SP was highly variable and was not altered following FPI (Fig. 5*D*).

These results indicate that CA1 pyramidal cells and interneurons are less able to synchronize to oscillatory SR synaptic input after FPI, whether because of decreased synaptic input itself, or because of an altered response, possibly contributing to the broad high-frequency power losses observed in SP after FPI. More importantly, loss of the precise timing relationship between CA1 synaptic inputs and CA1 pyramidal cell firing patterns could have significant implications for the ability of the hippocampus to encode information.

### TBI leads to a reduction in coordinated rhythmic inhibition of medial septal neurons by CA1

The medial septum is thought to play an important role in the generation and maintenance of hippocampal theta oscillations. Conversely, during periods when CA1 activity is dominated by sharp-wave ripples, feedback projections to the MS have been shown to inhibit MS cell firing (Dragoi et al., 1999). We would therefore expect synchronization of MS neuron firing with both CA1 theta oscillations and sharp-wave ripples. Thus, if our observed oscillation in SR under anesthesia indeed reflects rhythmic sharp waves, we would expect MS neurons to be rhythmically inhibited by it. Given reports of reduced theta power after TBI as well as our observed reduction in SR synaptic input after FPI, we asked whether there was either reduced synchronization of MS neuron firing with CA1 anesthesia theta or a reduction in the modulation of the MS by CA1 SR oscillatory input (Fig. 6).

A total of 147 and 182 medial septal cells were isolated in the sham and injured groups, respectively [sham group, 25 (range, 15–50) cells per animal; vs injured group, 33 (range, 17–54) cells per animal; Mann–Whitney, *p* = 0.45). As different neuron subtypes within the MS are known to play different roles in the regulation of hippocampal activity, we used a two-step process consisting of established waveform shape criteria adapted from King et al. (1998) followed by a firing rate cutoff to divide the isolated MS cells into putative subtypes: cholinergic versus GABA/Glu populations (Fig. 6*A–E*). Examples of putative GABA/Glu and putative cholinergic cells are shown in Figure 6*A*; 82 putative GABA/Glu cells were isolated in the sham group [median per rat: 14 (range, 10–22), while 143 were isolated in the injured group (median per rat: 26 (range, 16–40)], representing a significant increase in the number per rat (Fig. 6*G*; Mann–Whitney test, *p* = 0.032). Thirty-six putative cholinergic were isolated in the sham group [median per rat: 6 (range, 0–14)] and 24 in the injured group [median per rat: 6 (range, 1–7)], which did not differ significantly between groups (Fig. 6*G*; Mann–Whitney test, *p* = 0.63). Similarly, the mean firing rate of putative GABA/Glu cells increased after injury (sham group, 5.30 ± 6.42 Hz; vs injured group, 8.46 ± 9.76 Hz; Welch’s *t* test, *p* = 0.0039), while that of putative cholinergic cells did not (sham group, 2.84 ± 1.64 Hz; vs injured group, 2.89 ± 1.55 Hz; Welch’s *t* test, *p* = 0.92; Fig. 6*H*). These findings suggest a selective disinhibition of MS GABA/Glu neurons after injury.

To assess the interactions between MS neuron firing and either anesthesia theta or the observed ∼10–15 Hz oscillatory synaptic input into CA1 SR, we first assessed the phase relationship between the trough of each oscillation and the spiking patterns of individual isolated MS neurons. Proportions of entrained cells after injury were preserved. Among putative GABA/Glu cells, we found that 24 of 82 (29%) in the sham group and 52 of 143 (36%) in the injured group were entrained to anesthesia theta (no difference: χ^2^ test, *p* = 0.06), while 28 of 82 (34%) in the sham group and 44 of 143 (31%) in the injured group were entrained to the CA1 10–15 Hz SR input (no difference: χ^2^ test, *p* = 0.73). Among putative cholinergic cells, we found 18 of 36 (29%) in the sham group and 11 of 24 (46%) in the injured group were entrained to anesthesia theta (no difference: χ^2^ test, *p* = 0.68), while 18 of 36 (50%) in the sham group and 9 of 24 (38%) in the injured group were entrained to the CA1 10–15 Hz SR input (no difference: χ^2^ test, *p* = 0.22). Among those putative cells that were significantly entrained, we measured the strength of that entrainment (mean resultant vector), finding no differences among putative GABA/Glu cells (sham group, 0.10 ± 0.09; vs injured group, 0.08 ± 0.06; Welch’s *t* test, *p* = 0.22), or among putative cholinergic cells (sham group, 0.11 ± 0.06; vs injured group, 0.12 ± 0.07; Welch’s *t* test, *p* = 0.75) to anesthesia theta. However, both putative cell subtypes lost strength of entrainment to the CA1 10–15 Hz SR input after injury (GABA/Glu: sham group, 0.06 ± 0.06; vs injured group, 0.04 ± 0.04; Welch’s *t* test, *p* = 0.04; cholinergic: sham group, 0.09 ± 0.07; vs injured group, 0.05 ± 0.03; Welch’s *t* test, *p* = 0.005; Fig. 6*I*). These results suggest that MS neurons are indeed synchronized both to CA1 anesthesia theta oscillations and sharp wave-like oscillations, but while anesthesia theta synchronization appears preserved after injury, sharp wave-like oscillation synchronization is impaired.

If the observed ∼10–15 Hz SR oscillation in CA1 indeed represents rhythmic sharp wave-like input from CA3, we would expect the oscillation to drive MS neuron firing patterns and not the reverse. To test this, we computed the MS spike-triggered average of the CA1 LFP for each significantly entrained putative MS cell. The averaged spike-triggered averages for both the putative GABA/Glu and cholinergic cell subgroups revealed a robust ∼11 Hz oscillation with the expected maximal amplitude and CSD sink within SR and a sharp phase reversal from SO to SR just before the MS neuron action potential in the sham group. This oscillation was severely attenuated in the injured group (Fig. 6*J*). To quantify this, we measured the difference between the maximum positive voltage deflection and the maximum negative deflection in SR of the averaged spike-triggered averages, confirming a significant reduction in the ∼11 Hz oscillation occurring just before the action potential in both GABA/Glu (sham group, 162.0 ± 146.6 μV; vs injured group, 95.6 ± 114.1 μV; Welch’s *t* test, *p* = 0.048, Fig. 6*K*, top) and cholinergic (sham group, 222.7 ± 174.5 μV; vs injured group, 87.1 ± 51.0 μV; Welch’s *t* test, *p* = 0.0059; Fig. 6*K*, bottom) MS cells after injury. These results suggest a loss of modulation of MS neuron firing patterns after injury by CA1 feedback driven by the observed CA1 SR oscillation.

As the projections from CA1 to MS are known to inhibit the firing of MS neurons time locked to the maximal coordinated CA3 input into CA1 (maximum negative deflection in the sharp-wave LFP; Dragoi et al., 1999), we explored whether our observed modulation of MS neuron firing patterns by ∼10–15 Hz oscillatory CA1 SR input was inhibitory. We calculated the average PSTH in each group for all MS neurons significantly modulated by the observed 10–15 Hz CA1 SR input triggered off the trough of that oscillation. In the sham group, this revealed a significant inhibition of the average MS neuron firing probability ∼14 ms after the maximal negative deflection (trough) of the CA1 SR oscillation (Fig. 6*L*, left). This inhibitory influence was not detectable in the injured group PSTH (Fig. 6*L*, right).

Together, these data suggest that while the MS maintains synchronization with CA1 anesthesia theta, it may lose coordinated inhibition from rhythmic CA1 sharp wave-like oscillations after injury. Increased numbers of detected putative GABA/Glu (but not cholinergic) cells as well as their increased firing rates after injury suggest they may be preferentially disinhibited. Lack of observed MS structural pathology suggests these changes may be a consequence of reduced or dyscoordinated hippocamposeptal projections into MS, rather than an injured MS network.

## Discussion

TBI can lead to profound cognitive dysfunction both acutely and chronically following even mild injury (Hoge et al., 2008; Monti et al., 2013; Maas et al., 2017; Wilson et al., 2017). The hippocampus is often identified as a potential substrate for this disruption as well as a potential target for neuromodulatory treatment because of its prevalent role in cognition and memory, and its demonstrated vulnerability in TBI (Santhakumar et al., 2001). Explanations for hippocampal-dependent dysfunction range from synaptic imbalance (Howard et al., 2007) to changes in coding and firing properties in remaining neurons in the hippocampal network (Wolf and Koch, 2016), such as reduced spatial specificity of place fields (Broussard et al., 2020). Several studies in the intact rodent brain after TBI have shown theta band specific-power losses in the hippocampus (Fedor et al., 2010; Lee et al., 2013, 2015), an intriguing finding given that theta oscillations are thought to play a central role in memory encoding (McNaughton et al., 2006a,b). However, Paterno et al. (2016) have reported only broadband power losses in the hippocampus that offer an alternate explanation for theta decreases, postulating that broadband power reflects either the magnitude or synchrony of local multiunit activity. An important question is therefore whether oscillatory power losses in the intact brain after TBI are anatomic layer specific and frequency band specific, and therefore a reflection of changes in distinct synaptic inputs and/or local firing properties, as well as whether the modulation of single-neuron firing patterns by these oscillations is impaired. At 7 d following TBI, we found the following: (1) that CA1 power changes after TBI are anatomic layer specific and frequency band specific, revealing that low-frequency power losses under anesthesia are localized to the dendrites in SR, potentially reflecting reduced oscillatory input from CA3, while high-frequency power losses are confined to the perisomatic region, likely reflecting altered firing patterns and local interneuronal activity in response to synaptic input; (2) that theta oscillation power is unchanged under deep isoflurane anesthesia; (3) that isolated single CA1 neurons are able to maintain normal firing rates despite reduced oscillatory input to SR, yet are unable to maintain proper synchronization to that input; and (4) that coordinated inhibition of isolated single MS neurons by the observed dominant CA1 SR oscillation is impaired after injury, potentially reflecting a loss of inhibitory feedback from CA1 to the MS during sharp-wave ripples.

### TBI leads to loss of CA1 synaptic input

The SR of CA1 is the principle target of CA3 pyramidal cell projections via the Schaffer collaterals. We found a loss of low-frequency oscillatory power confined to SR, consistent with our observation of CA3 neurodegeneration and Schaffer collateral axonal pathology 7 d after injury. During consummatory behaviors and non-rapid eye movement (REM) sleep, episodes of synchronized input into SR from CA3, termed sharp waves, organize local CA1 neuronal activity into bursts, a process thought to be important for memory decoding (Buzsáki, 1986, 2015). Synchronized input into SR from CA3 has been observed under deep isoflurane anesthesia in the form an ∼10–15 Hz oscillation with the same anatomic pattern in the CA1 LFP and with the same synchronization of CA1 neuronal activity, as observed with sharp-wave events in the nonanesthetized rat. Further, 2–5 Hz oscillations that follow the anatomic pattern of awake theta oscillations (here termed anesthesia theta) can be observed under light isoflurane anesthesia (Lustig et al., 2016). Whether this pattern is the equivalent of theta observed during exploratory behavior or, for example, REM sleep in terms of the driving inputs that shape it, requires further study.

We found ∼10–15 Hz rhythmic SR input dominated the CA1 LFP in our anesthesia recordings in both groups, but was significantly reduced in power after TBI, while the brief episodes of anesthesia theta LFP patterns were unchanged. Loss of CA3 neurons and accompanying alterations in CA3–CA1 synaptic activity are a core feature of the lateral FPI model (Smith et al., 1991, 1997; Hicks et al., 1993; Grady et al., 2003), as well as other TBI models (Baldwin et al., 1997; Scheff et al., 2005; Norris and Scheff, 2009; Wolf et al., 2017). Thus, a loss in SR synaptic input after TBI could very well reflect reduced, or disrupted, CA3 inputs via Schaffer collaterals. The present study provides the first *in vivo* link between the known loss of CA3 activity after FPI and oscillatory power changes in CA1 in the intact brain.

The apparent preservation of theta power in our data is noteworthy, particularly given that we observed axonal pathology of all major inputs to the hippocampus, including into the SL-M and dentate molecular layer, which receive major inputs that drive theta oscillations (Buzsáki, 2002). This apparent discrepancy could reflect a post-TBI compensatory mechanism whereby remaining intact inputs have “retuned” to maintain normal levels of synaptic activity. This may suffice to preserve observed anesthesia theta during low theta states, such as deep isoflurane anesthesia (Lustig et al., 2016), but could manifest as reduced theta power during high-theta states, such as exploratory behavior. Our findings are therefore not inconsistent with previous reports and underscore that others’ observations of theta power loss after TBI are likely state dependent (Fedor et al., 2010; Lee et al., 2013, 2015). Variations in impact size and location on the skull, rodent species, and severity of injury reported in the FPI literature may also explain the variation in findings. Further, isoflurane itself can reduce neuronal firing rates and oscillatory power as well as shift the balance between inhibitory and excitatory current flow. We chose to perform this experiment under isoflurane anesthesia to facilitate the simultaneous recording of single-unit activity in both CA1 and MS. Therefore, our findings of specific power losses, firing pattern changes, or inhibitory activity under a constant anesthetic state require confirmation in the awake animal during the appropriate behavior. Nonetheless, alterations in coupling between coordinated synaptic inputs, as reflected in LFP oscillatory activity and local neuronal firing patterns, can provide important insights into the hippocampal network alterations that occur after TBI.

### Local CA1 neurons maintain normal firing rates, but cannot synchronize properly to stratum radiatum input after TBI

Coordinated synaptic input into SR from CA3 plays a major role in organizing local CA1 pyramidal cell and interneuron firing patterns during states of consummatory behavior and non-REM sleep, and serves as a possible mechanism of local information encoding and distribution to efferent structures (Carr et al., 2011; Buzsáki and Moser, 2013). One might expect decreased individual CA1 neuron activity in the face of a reduction in the dominant oscillatory input after TBI, yet we found no changes in mean firing rates of isolated CA1 neurons, consistent with previous reports (Eakin and Miller, 2012; Munyon et al., 2014). However, we did find that isolated CA1 pyramidal cells could no longer synchronize properly to this dominant CA3 input. This is particularly significant as states dominated by CA3-derived sharp waves are the most synchronous states in the intact hippocampus, with the highest firing probability of CA1 neurons occurring during the negative peak of the sharp wave observed in the CA1 LFP (Buzsáki, 2015). If local CA1 neurons are unable to appropriately respond to a major input into SR, their ability to follow more complex oscillatory patterns, also critical for memory encoding and decoding, may be disrupted.

We suggest that since CA1 neurons were able to maintain normal firing rates, yet unable to synchronize appropriately to oscillatory SR inputs, a compensatory mechanism may have been able to adjust for firing rate. Whether intrinsic or synaptic, this may come at the cost of maintaining more subtle input/output relationships. If this is a homeostatic plasticity mechanism whereby the remaining inputs are strengthened to maintain consistent output, this may in turn lead to a loss of the ability of neurons to synchronize to their previously dominant input and affect normal computation in the dendritic arbor, such as ensemble organization within a sharp-wave ripple event (see below). Consistent with this idea, we have previously reported a mismatch between lost fiber volleys in the Schaffer collateral pathway and decreased CA1 EPSPs 1 week post-TBI in a large-animal model, leading us to speculate that a form of homeostatic plasticity may be responsible (Turrigiano, 1999; Dinocourt et al., 2011; Wolf et al., 2017).

### Local CA1 high-frequency oscillations are decoupled from stratum radiatum input while synchronizing local neuron firing to a broader, nonspecific frequency range after TBI

In addition to individual CA1 neuron action potentials, we also observed discrete high-frequency oscillatory events in the pyramidal cell layer that were coupled to the negative peak of the observed ∼10–15 Hz oscillatory input into SR. CA3-derived sharp-wave input into SR drives the occurrence of brief, high-frequency oscillations such as fast gamma and ripple oscillations in the pyramidal cell layer via local interactions between pyramidal cells and interneurons, resulting in a temporal coupling of sharp waves and these high-frequency events (Ylinen et al., 1995; Klausberger and Somogyi, 2008). SPW-Rs are known to organize ensembles of CA1 pyramidal cells into a particular firing sequence that recapitulates previous experiences, or future actions, and as such are thought to be critical for hippocampal-dependent memory (Carr et al., 2011). Although poorly defined under anesthesia, the observed perisomatic high-frequency oscillations in this study had frequencies that encompassed and extended beyond those expected of true ripples. Whether derived from similar mechanisms or not, the high-frequency oscillations observed in this study may represent both CA1 synchronized multiunit activity and local pyramidal cell–interneuronal interactions (Schomburg et al., 2012). We observed a power loss after injury within the extended ripple frequency range (190–280 Hz) in the pyramidal cell layer. We also observed a phase–amplitude decoupling of these high-frequency oscillations with the observed low-frequency sharp wave-like (∼10–15 Hz) SR synaptic input, possibly representing rhythmic input from CA3 (Lustig et al., 2016). To the extent that discrete high-frequency oscillation events under anesthesia are generated by the same or similar mechanisms as true ripples, the observed decoupling and power losses after injury could reflect impaired pyramidal cell–interneuron interactions. Loss of coordinated CA3 inputs could also directly contribute to these findings, whether because of the observed Schaffer collateral pathology or CA3 neuronal injury, as reduced numbers of synchronized bursting CA3 pyramidal cells have been shown to result in fewer SPW-R events and reduced ripple power in CA1 (Csicsvari et al., 2000).

In addition to synchronizing with oscillatory synaptic input, local CA1 neurons also synchronize to the phase of ripple oscillations (Buzsáki, 2015). Alternatively, fast oscillations (>200 Hz) may be due directly to synchronized local action potentials (Schomburg et al., 2012). It is therefore not surprising that we observed entrainment of putative CA1 pyramidal cells to high frequencies with a peak at 225 Hz in sham animals. Following TBI, the range of entrainment broadened with significant increases in entrainment below (71–176 Hz) and above (>280 Hz) the classic ripple frequency range. One explanation could be that local pyramidal cell–interneuron interactions responsible for ripple generation are impaired, leading to a “detuning” of pyramidal cell high-frequency entrainment within preferred ripple frequencies and allowing other mechanisms of fast oscillation generation to dominate. These may then entrain neurons across a wider and potentially inappropriate frequency range, such as high gamma oscillations, pathologic high-frequency oscillations, or default to current directly from pyramidal cell action potentials (Bragin et al., 1999; Sullivan et al., 2011; Schomburg et al., 2012). These results imply an impaired ability to maintain context-specific CA1 neuron ensemble organization after injury. However, the loss or dysfunction of SPW-R complexes in the awake behaving animal remains to be demonstrated.

### TBI leads to impaired hippocampal inhibitory modulation of the medial septum

Generation and maintenance of hippocampal theta requires rhythmic input from the MS (Buzsáki, 2002). Experimental loss of theta power along with spatial memory performance can be rescued by MS stimulation (McNaughton et al., 2006b). However, the functional coupling between these structures is both reciprocal and state dependent (Dragoi et al., 1999; Borhegyi et al., 2004). For example, the majority of GABAergic MS neurons alter their response to synaptic inputs depending on the current hippocampal state (Toth et al., 1993; Dragoi et al., 1999; Borhegyi et al., 2004).

We found no evidence to support direct injury to the MS in the number of isolated MS cells, histologic inspection, or a reduction in overall firing rate. However, our histologic examinations were performed 7 d postinjury using markers that only detect cells or axons that are actively within the process of degeneration. As such, analyses will not identify cells or axons that may have degenerated acutely in the preceding days, the loss of which may influence electrophysiological findings.

After injury, we found intact coupling between MS neurons and CA1 anesthesia theta, but a loss of coordinated modulation of MS cells by the dominant CA1 10–15 Hz oscillation in SR. This loss of MS neuron modulation appears to be a loss of inhibition and is coupled with both an increase in firing rate and an increased number of isolated GABAergic/glutamatergic MS neurons. As identification of putative single neurons from extracellular recordings requires adequate numbers of spontaneous action potentials (Thompson and Best, 1989), the detection of more MS neurons after injury likely reflects the overall increase in firing rate after injury rather than a true increase in the number of neurons postinjury. This finding may reflect loss of GABAergic feedback from CA1 stratum oriens interneurons to the MS (Müller and Remy, 2018). Disruption of HC modulation of the MS after TBI could reflect downstream consequences of the observed loss of synaptic activity into CA1 after injury, injury/dysfunction of CA1 interneurons, synaptic changes within the MS, acute loss of MS neurons that have already been cleared (see Discussion above), or a combination of these effects. Given the reciprocal relationship between the structures, our findings have implications for other HC states, including the proper generation and maintenance of theta during exploratory behavior.

In summary, we have provided the first *in vivo* evidence of anatomic layer-specific and frequency-specific changes in hippocampal oscillatory power 7 d after TBI, reflecting both losses of coordinated synaptic input into CA1, as well as a loss of coordinated local CA1 neuronal activity. These findings provide one possible link between previous reports of CA1 power loss and the existing substantial structural and *ex vivo* electrophysiological evidence of CA3 neuronal loss as well as disrupted CA3–CA1 interactions after FPI, the most well established rodent model of TBI. Further, these findings provide the first *in vivo* evidence of (1) the failure of the principle output neurons of the hippocampus to properly synchronize to their synaptic inputs after TBI, a mechanism fundamental to hippocampal memory encoding and decoding; and (2) the failure of the hippocampus to exert normal modulation of an external structure, the medial septum, which may have implications for the proper state-dependent maintenance of hippocampal theta oscillations. We have found that the firing rates of CA1 neurons under isoflurane anesthesia appear unchanged, further supporting the hypothesis that the prominent dysfunction of the remaining intact network is in the ability to coordinate inputs and oscillations normally bound together that organize activity in the postsynaptic neurons. If confirmed in the behaving animal, these data could suggest that the normal SPW-R mechanism arising from CA3–CA1 interactions might be disrupted following TBI or neurons may not organize correctly in response. The ability to consolidate memory episodes during both waking and sleeping, as well as potentially distribute them beyond the hippocampus, may therefore be impaired. This may also explain the inability of the rats to recall the learned position of the platform in the water maze following injury. Finally, the preserved capacity of the remaining CA1 neurons to achieve normal firing rates 7 d post-TBI implies that therapeutic modulation of these networks may still allow for proper encoding of spatial and other cognitive information in the hippocampus, potentially using closed-loop strategies that are responsive to the current network state (Heck et al., 2014; Ezzyat et al., 2017).

## Synthesis

Reviewing Editor: Katalin Toth, Universite Laval Decisions are customarily a result of the Reviewing Editor and the peer reviewers coming together and discussing their recommendations until a consensus is reached. When revisions are invited, a fact-based synthesis statement explaining their decision and outlining what is needed to prepare a revision will be listed below. The following reviewer(s) agreed to reveal their identity: Viktor Varga, Gene Gurkoff.

This is a timely study on the impact of TBI on oscillatory behaviour in the hippocampus. While both reviewers agree that the central question and the presented findings are important and novel, they both made several constructive comments that could help the authors to improve he quality of this manuscript.

Rev. #1:

The manuscript fits in the line of previous studies about the effect of traumatic brain injury (TBI) on neuronal oscillations. As duly cited, preceding studies described TBI effect on the spectral characteristics of various oscillations. The current manuscript attempted to go further by analyzing layer-specificity of TBI-triggered alterations and the changes TBI induces in the interaction among oscillations. It also widened the scope of analysis by including the medial septum as a potential source through which TBI effects can lead to altered CA1 activity.

The Authors studied the alterations of oscillations and their interactions in the CA1 subfield of the hippocampus in response to mild traumatic brain injury (TBI) in anesthetized rats. As duly acknowledged, measuring the effect of TBI under anesthesia has limitations but the results can be deemed important additions to our knowledge about the effects of head impacts on brain function. However, the analysis can be expanded and the presentation of some of the results should be refined. My concerns are as follows:

The Authors focus mostly on the alterations of activity in the Schaffer collaterals' termination zone in the stratum radiatum albeit the degeneration of all the major hippocampal input pathways is reported. Moreover, the amplitude of the slow theta CSD seems to be higher in the lacunosum moleculare (S-LM, Figure 1C and E 2-5 Hz CSD) of injured animals relative to sham but theta was stated to be unchanged. One technical reason can be that only few channels close to the tip of the probe reached the S-LM therefore it was spatially undersampled. The APP immunostaining also points to the S-LM as the locus of the most profound anatomical change. Therefore, showing convincingly the lack of reduction or augmentation of low frequency oscillations in the S-LM would be important and the discrepancy between the anatomy and physiology should be explained in the Discussion.

The anatomical defects have to be quantitatively described. For example, the number of Fluoro-jade C positive neurons in injured versus sham control and the density of APP-labeled terminals in the hippocampal formation should be compared and presented with the data of both the individual animals and group statistics. Loss of certain neuron types, as suggested by the electrophysiological data, would need to be verified by a double immunostaining with Fluoro-jade C and interneuron markers.

There is no supporting data or image for the claim about the lack of injury of the medial septum. If, as hypothesized, a reduced hippocampo-septal feedback was responsible for the higher activity of MS neurons, a Fluoro-jade C-staining in the stratum oriens is expected to be detectable accompanied by some degenerating axons in the MS. Nevertheless, a photomicrograph about the septal region is necessary.

While the coupling of unit activity to oscillations was analyzed in detail, interaction among neurons was not dealt with, for example by cross-correlation analysis. Since discoordination of activity is assumed to be a key consequence of TBI and the underlying mechanism of the observed decoupling of oscillations across layers, this analysis would be especially important.

The separation of pyramidal cells and interneurons based on spike width, autocorrelation first moment and firing rate looks a bit arbitrary. Particularly the firing rate of interneurons is too low (the lower limit is usually 5 Hz but there are low firing interneurons e.g. NPY-expressing ones). A higher firing rate threshold is clearly visible as a gap just below 5 Hz on the 3-D scatterplot of Figure S3 panel A. The autocorrelogram on Figure 4B can also belong to a pyramidal cell. Thus, cell classification should be reanalyzed.

On the cross-frequency coupling plots there’s an association of sub-10 Hz and 60-70 Hz with increased magnitude in the injured animals but this was not detailed in the Results. What can be the source of this sub-10 Hz coupled mid-gamma-like oscillation?

There are multiple neuron types in the MS with radically different firing characteristics (and functions) therefore, distinguishing the effect of TBI on the various MS neuron types should be examined. Synchronization of activity is linked to the GABAergic, fast and rhythmic neurons of the MS therefore, the possible role of these cells in the loss of synchrony could be analyzed by revealing the correlation between cell type (based on firing rate, spike width and theta rhythmicity) and TBI effect. For example, it is possible that fast spikers (putative GABAergic) are more affected than slower (putative cholinergic) neurons.

A minor comment about the occurrence of sharp waves: in the awake animals it occurs during consummatory behaviors and not when the animal is in a still alert state (when type 2 theta is more prominent).

Diameter of the Morris water maze pool was not given (only XX).

Rev. #2:

For many years there have been documented changes in anatomy and behavioral outcome in models of traumatic brain injury with very limited data investigating changes in the neural network. Over the last few years a small number of manuscripts have evaluated changes in the oscillatory network. However, this is the first manuscript to use multiple arrays, to look at inputs and outputs, to look at spike-wave coupling and feedback between the hippocampus and septum. So this work represents a significant advancement compared to previous publications in the field.

In the manuscript entitled “Traumatic Brain Injury Preserves Firing Rates But Disrupts Laminar Oscillatory Coupling and Neuronal Entrainment in Hippocampal CA1” the authors demonstrate a change in oscillatory activity, spike wave coupling and CA1-MS feedback in following the rodent fluid percussion injury model. These findings add significantly to a small but growing field related to neural activity following TBI. On the whole I find the data exciting and informative. While recordings under anesthesia do not allow the reader to put the electrophysiology in the context of specific behavioral deficits, the experimental design is justified by the authors. All of the following comments are minor, and several are simply suggestions for readability.

Minor Comments:

Comment: In the methods the diameter of the pool is described as “xx m in diameter and 50 cm tall...”

Comment: In the methods you describe the temperature of the water as 18{degree sign}C (64{degree sign}F). This is a cold pool and could have a substantial effect on animal’s behavior particularly in the first days post-injury when they are still in recovery. Nothing you can change here, but if you look at the greater literature I think you will find that this is a cold pool.

Comment: For fluid percussion surgery the craniotomy is defined as over left parietal cortex midway between bregma and lamda? How far off midline was the center of the craniotomy and how large was the craniotomy?

Comment: For methods related to fluid percussion, there is a lot of variability between devices. Please let the reader know whether FPI=2.3 ATM corresponds to a typical mild, moderate or severe injury in your laboratory. In our lab a 2.3 ATM force would result in 100% mortality as an example.

Comment: As the author suggests, these findings follow several manuscripts related to oscillations and behavior in rats and mice. One unique aspect of this paper is that the data was collected during wakeful hours as the rats were on a reverse light cycle. This is both interesting and important and could warrant a line or two in the discussion as a strength.

Comment: The experimental design is a little quirky in that behavior was analyzed within two days after injury and then electrophysiology was analyzed on day 7 and under anesthesia, so ephys findings do not directly correspond to any behavior per say. These are important differences, and the interpretation of electrophysiology in anesthetized as opposed to awake and behaving rats is different. This is mostly covered in the discussion - but perhaps a little extra clarity oh the difference between anesthetized and awake theta - and perhaps even awake and behaving (which is different from awake and restrained or awake and lethargic).

Comment: There is minimal description of the analysis of histological findings in the methods. How many sections? What method of quantification? Specific coordinates? When I read the results there were really nice images and it is clear that there is APP and FJC staining, but there are no numbers associated with the findings. Analysis and interpretation needs to be clarified. Clearly if this is an all or none (or nearly none) effect then stereology would be over-kill, but some sort of description is needed.

Comment: In our hands, we cannot test our moderate to severe FPI in the MWM until at least 3 days after injury and typically wait at least 1 week. There is a lot of variability in the field over what is considered mild, moderate and severe. Clearly there is a lot of histological damage - so this comment is not to suggest that the injury was less than severe. Perhaps it is best to clarify that consistent with moderate-to-severe injury based on the specific FPI paradigm?

Comment: For Figure 1, do the spectrograms B&C and E&F correspond to a time window in the traces in A&D?

Comment: In the section entitled “FPI reduces coupling of Local CA1 Stratum...” there is a mixture of methods, results and discussion. I recognize that some of the methods/discussion may be necessary to contextualize the results, but even a little streamlining might reduce the bulk of this section. Just a suggestion.

Comment: A similar suggestion for the following section where a lengthy discussion to contextualize the data exists in the results section. I understand why it is here, but at the same time could readability be improved by tightening the results and moving to discussion? Again, just a suggestion.

Comment: I found the paragraph entitled “Injured Neurons Lose Proper Entrainment...” a little confusing. There is a subtle change from the first paragraph talking about entrainment and the second paragraph talking about degree of entrainment. The transition to the specific phase was cleaner. Tweaking a few words should make this more clear.

Comment: At the end of the TBI Leads to Loss of CA1 Synaptic Input section of the discussion, the authors try to put their data into context and work hard not to contradict the prior work of their colleagues. One thing that the author does not describe in this section is that the Fedor/Lee papers uses a slightly different model of FPI as compared to the current paper and that the Paterno paper evaluated oscillations in mice. The Eakin/Miller group had a more mild injury. This is in addition to the fact that different states of consciousness and different behaviors were used. I think it is important to also mention the variance in injury as well as species because these are relevant ideas to consider as we plan future studies and continue to interpret these data in the proper context.

Comment: To follow the previous comment, even though there is variance in the data to whether it is 5 Hz, 10 Hz or broadband changes, each of the findings suggests that there is a disruption in neural activity concomitant with cognitive dysfunction - which is also important to point out.

## Author Response

Response to Reviewers

We thank the reviewers for their efforts as well as their insightful comments. We believe the manuscript is much improved with these revisions, and have attempted to address each comment below as well as in the manuscript where appropriate.

Reviewer #1

Comment #1: “...the amplitude of the slow theta CSD seems to be higher in the lacunosum moleculare (S-LM, Figure 1C and E 2-5 Hz CSD) of injured animals relative to sham but theta was stated to be unchanged. One technical reason can be that only few channels close to the tip of the probe reached the S-LM therefore it was spatially undersampled. The APP immunostaining also points to the S-LM as the locus of the most profound anatomical change. Therefore, showing convincingly the lack of reduction or augmentation of low frequency oscillations in the S-LM would be important and the discrepancy between the anatomy and physiology should be explained in the Discussion.”

Response: We agree that the CSD plots of the 2-5 Hz oscillation could suggest that oscillatory power is increased in the injured group relative to the sham group, but that this is not captured in our formal group comparisons. We have addressed this in two ways. First, we have clarified the figure legend to indicate that the CSD/LFP traces in Figure 1 are example tracings from a single sham and a single injured rat, which may not reflect overall CSD power changes between groups. Second, as suggested by the reviewer, we may not have captured the true change in slow theta power, as our aligned, common set of 25 (of all 32) recording channels did not cover the SL-M, which is where the greatest theta power would be expected. We therefore identified the subset of sham (n=3) and injured (n=4) rats that had probes sufficiently deep (at least 100 microns below the bottom-most common channel) to capture the SL-M and compared slow theta (2-5 Hz) power on the bottom, aligned channel. This confirmed no power differences. The text of the Results section has been updated to reflect this additional analysis.

With regard to the histological findings, we agree that APP pathology was observed within deeper layers of the dorsal hippocampus in both the SL-M and the molecular layer. Discrepancies between SL-M axonal pathology and preserved SL-M slow theta band synaptic input could suggest a compensatory mechanism at work, whereby input oscillatory activity coming from entorhinal cortex, for example, via remaining intact axonal projections have “re-tuned” in order to maintain normal levels of activity in the face of damage. This may suffice to preserve slow theta inputs in a low theta state such as deep isoflurane anesthesia, but could manifest as reduced theta drive during high theta behavioral states. The Discussion has been revised accordingly.

Comment #2: “The anatomical defects have to be quantitatively described. For example, the number of Fluoro-jade C positive neurons in injured versus sham control and the density of APP-labeled terminals in the hippocampal formation should be compared and presented with the data of both the individual animals and group statistics. “

Response: We thank the reviewer for raising this point. However, the intent of the histological analyses within this report were to provide an overview of the distribution of pathology observed within the hippocampus and associated structures within this model. A specific subset of animals were dedicated to histological assessment only, since the process of electrode insertion can result in confounding pathology, particularly the often subtle axonal swellings observed. As such, since histopathology was not performed in the same animals as electrophysiological examination, we feel highly quantitative data within individual animals will not add additional useful information for interpretation. Moreover, the quantification of axonal pathology with respect to connectivity offers particular technical challenges that would be difficult to address with the material available (see Weber et al, 2018). We have however amended the results section to provide additional information regarding the presence of pathology within each subregion, including the number of animals where it was observed. Additional subregions as requested below are also now included.

Comment #3: “Loss of certain neuron types, as suggested by the electrophysiological data, would need to be verified by a double immunostaining with Fluoro-jade C and interneuron markers.”

RESPONSE: We agree with the reviewer that detailed analyses of the neuronal subtypes in relation to cell death would be of interest in the context of these electrophysiological data. However, this would require detailed quantification of multiple interneuron / cell death markers, ideally assessed over a temporal course, and in parallel with electrophysiological examinations within individual animals. While we believe this is beyond the scope of the current study, we have initiated these experiments as part of ongoing work within our group.

Comment #4: “There is no supporting data or image for the claim about the lack of injury of the medial septum. If, as hypothesized, a reduced hippocampo-septal feedback was responsible for the higher activity of MS neurons, a Fluoro-jade C-staining in the stratum oriens is expected to be detectable accompanied by some degenerating axons in the MS. Nevertheless, a photomicrograph about the septal region is necessary.”

RESPONSE: We thank the reviewer for this suggestion and have now included additional description and photomicrographs of findings in the medial septum and stratum oriens which now reads “Within the medial septum there was no evidence of any axonal pathology or fluoro-jade-C positive cells in any injured animals (Figure 2, P). Ab246 immunoreactivity was also absent in 3 injured animals, with the remaining injured animal displaying just a single positive cell (Figure 2, Q and R). There was similarly no evidence of overt cell death within stratum oriens.”

An important caveat to interpretation of the above, and indeed all reported histological findings, is that examinations were performed at 7 days post-injury and markers permit detection of cells / axons actively within the process of degeneration. As such, analyses will not identify cells that may have degenerated acutely in the preceding days, the loss of which may influence electrophysiological outcome. A note to this effect has been added to the discussion.

Comment #5: “While the coupling of unit activity to oscillations was analyzed in detail, interaction among neurons was not dealt with, for example by cross-correlation analysis. Since discoordination of activity is assumed to be a key consequence of TBI and the underlying mechanism of the observed decoupling of oscillations across layers, this analysis would be especially important.”

Response: We whole-heartedly agree that the interactions between individual neurons is crucial for hippocampal function and, indeed, ultimately underlies the generation of oscillatory patterns and potentially cognitive function. We anticipate discoordination within and among neuronal ensembles after TBI and indeed we are currently pursuing these analyses in awake behaving animals. The behavior of CA1 neuronal ensemble synchrony is well-described in the awake behaving state: for example, cross-correlation between pyramidal cells drops during high theta states and rising during sharp wave ripple states. However, under anesthesia the expected behavior and significance of neuron-neuron synchrony beyond what can be explained by their interactions with driving oscillatory inputs is less clear. Formation of information-rich neuronal ensembles such as memory “engrams” may not occur at all under such a uniform state as anesthesia. For this reason, we have chosen to pursue these types of analyses only in our awake behaving data, which is currently in progress.

Comment #6: “The separation of pyramidal cells and interneurons based on spike width, autocorrelation first moment and firing rate looks a bit arbitrary. Particularly the firing rate of interneurons is too low (the lower limit is usually 5 Hz but there are low firing interneurons e.g. NPY-expressing ones). A higher firing rate threshold is clearly visible as a gap just below 5 Hz on the 3-D scatterplot of Figure S3 panel A. The autocorrelogram on Figure 4B can also belong to a pyramidal cell. Thus, cell classification should be reanalyzed.”

Response: We very much appreciate the reviewer’s insights into CA1 cell subtype classification and have reworked our approach. In our previous method, putative single units were grouped based on their clustering in 3 dimensions, corresponding to spike width, autocorrelation first moment and firing rate. However, as pointed out by the reviewer, the firing rate did not turn out to provide any meaningful classification information. Our new approach is as follows:

First, as we did before, the anatomical location of each putative single unit was estimated as stratum oriens (SO), stratum pyramidale (SP), or stratum radiatum (SR). By definition, single units outside SP are not pyramidal cells and we therefore classified them as putative interneurons. Although some of these units had slower firing rates, there are interneurons that have slower firing rates, as pointed out by the reviewer, and they tend to be located outside of SP. Classic fast-spiking interneurons (for example, PV+ basket cells) are generally found in SP. Second, we calculated 3 features for all putative single units within SP: spike width, first moment of the autocorrelation and firing rate. We inspected the distribution of these features which revealed 2 natural clusters in each. We then made a simple division based on these clusters for each feature (see Figure 4B). Putative pyramidal cells were defined as those units with a spike width above the determined threshold, a first moment of the autocorrelogram below the determined threshold (equating to high burstiness) and a firing rate below the determined threshold (division was at 3.5 Hz). Using this more stringent approach we were only able classify 78/116 (67%) of sham and 59/86 (69%) of injured single units, compared to 99% in each group using our original method. Encouragingly, nearly all subsequent findings were maintained, or enhanced. We felt that the new method was superior given that it was able to use firing rate as a feature for classification, an important factor for defining interneurons, particularly in the SP. The fact that the firing rate cutoff was 3.5 Hz, potentially lower than what might be expected for fast-spiking interneurons, can be explained by our recordings being made under isoflurane anesthesia. The Methods and Results sections as well as figures have been appropriately revised.

Finally, we agree that the example putative interneuron (from the original classification scheme) in Figure 4 did not clearly demonstrate classic interneuron features. We have updated Figure 4 with an example putative interneuron using the new classification scheme.

Comment #7: “On the cross-frequency coupling plots there's an association of sub-10 Hz and 60-70 Hz with increased magnitude in the injured animals but this was not detailed in the Results. What can be the source of this sub-10 Hz coupled mid-gamma-like oscillation?"

Response: We agree that the apparent coupling of the phase of a ∼10 Hz oscillation in SR with the amplitude of a mid-gamma (∼60-70 Hz) oscillation in SP in interesting, as there is a known coupling of gamma oscillatory power to both CA3 inputs (low gamma, ∼30-40 Hz) and MEC inputs (∼80-100 Hz). The observed gamma oscillations could represent a blend of these responses, given the artificial anesthetic state. Although this coupling was indeed numerically higher in the injured group compared to the sham group, as suggested by the figure, there was no statistical difference, and we therefore chose not to comment on it in the text.

Comment #8: “There are multiple neuron types in the MS with radically different firing characteristics (and functions) therefore, distinguishing the effect of TBI on the various MS neuron types should be examined.

Synchronization of activity is linked to the GABAergic, fast and rhythmic neurons of the MS therefore, the possible role of these cells in the loss of synchrony could be analyzed by revealing the correlation between cell type (based on firing rate, spike width and theta rhythmicity) and TBI effect. For example, it is possible that fast spikers (putative GABAergic) are more affected than slower (putative cholinergic) neurons. “

Response: We are very appreciative of the suggestion and agree that neuronal subtypes within the MS play different roles in hippocampal function in the awake, behaving rat, particularly in maintaining theta and non-theta states. We have investigated numerous methods of classifying isolated MS single units into putative GABAergic and putative cholinergic cells. We calculated numerous features, including firing rate, spike width, and several characteristics of the autocorrelogram (burstiness, rhythmicity with respect to theta, etc) and looked at numerous combinations in an attempt to subtype the MS units, but no clear clustering could be found. Most reports of MS subtype classification are either in awake behaving animals, or under urethane anesthesia, under which theta oscillations can be observed. In addition, firing rates can be significantly diminished by anesthesia, which could have differential effects on different neuron subtypes. Our difficulties, therefore, may be related to isoflurane anesthesia and/or a low theta state. In one report from the O’Keefe lab (King et al, 1998) we found a classification scheme in awake behaving rats based entirely on waveform shape analysis that was originally reported in in vitro intracellular and juxta-cellular slice recordings (Matthews and Lee, 1991). Applying this scheme to our data yielded promising results, which we have detailed in Figure 6, as well as in the Results and Methods sections. Using this scheme, we found that putative GABAergic units appear to account for the increased firing rates observed in the MS after injury, with no change in cholinergic cells. We also found a higher proportion of the total MS units to be GABAergic after injury, which could further support disinhibition of hippocampal feedback, as these may represent more individual units that have firing rates above our detection criteria. However, we found no differences between cell types in proportions entrained to CA1 oscillatory activity (slow theta, or 10-15 Hz in SR) in either the sham, or injury groups. We therefore combined subtypes in subsequent entrainment analyses and figures.

Comment #9: “A minor comment about the occurrence of sharp waves: in the awake animals it occurs during consummatory behaviors and not when the animal is in a still alert state (when type 2 theta is more prominent).”

Response: We appreciate and agree with the clarification and have revised the text.

Comment #10: “Diameter of the Morris water maze pool was not given (only XX).”

Response: We much appreciate the reviewer catching this omission. The text has been revised to include the pool diameter, which is 2 meters.

Reviewer #2

Comment #1: In the methods the diameter of the pool is described as “xx m in diameter and 50 cm tall...”

Response: We thank the reviewer for noting this omission, and we have made the correction.

Comment #2: In the methods you describe the temperature of the water as 18C (64F). This is a cold pool and could have a substantial effect on animal’s behavior particularly in the first days post-injury when they are still in recovery. Nothing you can change here, but if you look at the greater literature I think you will find that this is a cold pool.

Response: We appreciate the insight and agree 18 degrees Celsius may be on the cooler side of what is reported. Going forward we will ensure that we achieve a more comparable ambient temperature.

Comment #3: For fluid percussion surgery the craniotomy is defined as over left parietal cortex midway between bregma and lamda? How far off midline was the center of the craniotomy and how large was the craniotomy?

Response: We thank the reviewer for pointing out the absence of detail. We have employed the lateral fluid percussion injury method as described by the McIntosh group at Penn in their original series of papers. The craniectomy was 5mm in diameter, centered between bregma and lambda in the AP dimension and over the left hemisphere such that the medial edge was almost at midline, placing the center of the craniectomy ∼2.5mm laterally. We have updated the Methods section appropriately.

Comment #4: For methods related to fluid percussion, there is a lot of variability between devices. Please let the reader know whether FPI=2.3 ATM corresponds to a typical mild, moderate or severe injury in your laboratory. In our lab a 2.3 ATM force would result in 100% mortality as an example.

Response: We agree that atmospheres of pressure correlate little with the severity of injury across laboratories. In our hands, 2.3 atm creates an injury consistent with previous reports in FPI of moderate to severe injuries. Our primary metrics for injury severity are the axonal and neuronal pathology we observed at 7 days post-injury, and performance on the Morris Water Maze on day 1. The details of these metrics are presented in the Results sections “Injury Physiology and Behavioral Deficits Confirm Moderate to Severe TBI” and “Histopathology Confirms Injury Severity ....”

Comment #5: As the author suggests, these findings follow several manuscripts related to oscillations and behavior in rats and mice. One unique aspect of this paper is that the data was collected during wakeful hours as the rats were on a reverse light cycle. This is both interesting and important and could warrant a line or two in the discussion as a strength.

Response: We agree that recordings during periods of wakefulness are critical to understanding hippocampal function, as the interaction between neuronal ensembles and synaptic inputs is highly dependent on the current behavioral and electrophysiological state of the animal. Whether generally being in the wakeful period of the circadian rhythm while under anesthesia can significantly influence hippocampal function is an interesting question, but one which we think is outside the scope of this study.

Comment #6: The experimental design is a little quirky in that behavior was analyzed within two days after injury and then electrophysiology was analyzed on day 7 and under anesthesia, so ephys findings do not directly correspond to any behavior per say. These are important differences, and the interpretation of electrophysiology in anesthetized as opposed to awake and behaving rats is different. This is mostly covered in the discussion - but perhaps a little extra clarity on the difference between anesthetized and awake theta - and perhaps even awake and behaving (which is different from awake and restrained or awake and lethargic).

Response: We agree with the reviewer that direct comparisons between behavioral deficits 1 day post-TBI and electrophysiological findings 7 days post-TBI are not meaningful and we have carefully avoided them. Our behavioral findings are primarily to validate that the FPI model in our hands results in the expected hippocampal-dependent memory deficits in a moderate to severe injury as previously reported.

We further agree that the interpretation of electrophysiological findings under anesthesia pose unique challenges that are distinct from awake behaving recordings. We appreciate the reviewer pointing out that the interpretation of a theta oscillation is one of those areas. In this study we rely on the pattern of phase precession and relative power differences in the observed 2-5 Hz frequency across the layers of the hippocampus under anesthesia to define “anesthesia theta,” following Lustig et al (2015) in arguing that this pattern has a striking resemblance to the pattern observed during exploratory theta activity in the awake rat. We have added a brief discussion of this in the Discussion.

Comment #7: There is minimal description of the analysis of histological findings in the methods. How many sections? What method of quantification? Specific coordinates? When I read the results there were really nice images and it is clear that there is APP and FJC staining, but there are no numbers associated with the findings. Analysis and interpretation needs to be clarified. Clearly if this is an all or none (or nearly none) effect then stereology would be over-kill, but some sort of description is needed.

Response: See response to Comment # 2 of Reviewer #1. Approximate AP levels of histological examinations in the hippocampus and medial septum have been added to the methods section.

Comment #8: In our hands, we cannot test our moderate to severe FPI in the MWM until at least 3 days after injury and typically wait at least 1 week. There is a lot of variability in the field over what is considered mild, moderate and severe. Clearly there is a lot of histological damage - so this comment is not to suggest that the injury was less than severe. Perhaps it is best to clarify that consistent with moderate-to-severe injury based on the specific FPI paradigm?

Response: The reviewer raises an important point regarding the variability in the field with regards to reporting of injury severity in the FPI model. Indeed, variable outcomes have been reported with seemingly similar input pressures during injury. Moreover, there are further considerations around how “moderate-severe” levels of injury in FPI in rats relate to clinical definitions of TBI severity.

As such, in the results section we have provided more specific and informative detail including that the model as described “recapitulates both focal and diffuse pathologies, including cortical contusion and widespread axonal pathology, commonly observed following moderate - severe TBI in humans. In addition, the extent and distribution of pathology was consistent with a degree of injury in the moderate-severe range for FPI (Smith et al, 1997).”

Comment #9: For Figure 1, do the spectrograms B&C and E&F correspond to a time window in the traces in A&D?

Response: We thank the reviewer for bringing up a subtle, but important point for clarification. The CSD pseudocolor maps in B & C, filtered to highlight the dominant 10-15 Hz oscillation in stratum radiatum, are taken from the same tracings depicted in A & D. However, the CSD pseudocolor maps in E & F depicting the 2-5 Hz oscillation in a theta-like laminar pattern are taken from a different segment of the recording from the same rats. This is important, as the reviewer may be pointing out, as the two rhythms were not observed to occur at the same time. We have updated the figure legend and Results section to make this clearer.

Comment #10: In the section entitled “FPI reduces coupling of Local CA1 Stratum...” there is a mixture of methods, results and discussion. I recognize that some of the methods/discussion may be necessary to contextualize the results, but even a little streamlining might reduce the bulk of this section. Just a suggestion.

Response: We thank the reviewer for the very helpful suggestions on improving readability of results and their interpretation that are complex. We have made an attempt to reduce the interpretation in the Results section and move relevant information to the Discussion section.

Comment #11: A similar suggestion for the following section where a lengthy discussion to contextualize the data exists in the results section. I understand why it is here, but at the same time could readability be improved by tightening the results and moving to discussion? Again, just a suggestion.

Response: See response to previous comment.

Comment #12: I found the paragraph entitled “Injured Neurons Lose Proper Entrainment...” a little confusing. There is a subtle change from the first paragraph talking about entrainment and the second paragraph talking about degree of entrainment. The transition to the specific phase was cleaner. Tweaking a few words should make this clearer.

Response: We thank the reviewer for the point of confusion and suggestions for improving readability. We have revised the text in this section for clarity.

Comment #13: At the end of the TBI Leads to Loss of CA1 Synaptic Input section of the discussion, the authors try to put their data into context and work hard not to contradict the prior work of their colleagues. One thing that the author does not describe in this section is that the Fedor/Lee papers uses a slightly different model of FPI as compared to the current paper and that the Paterno paper evaluated oscillations in mice. The Eakin/Miller group had a milder injury. This is in addition to the fact that different states of consciousness and different behaviors were used. I think it is important to also mention the variance in injury as well as species because these are relevant ideas to consider as we plan future studies and continue to interpret these data in the proper context.

Response: We agree completely with the reviewer and appreciate the suggestion to make these points in the discussion. We have added a brief discussion of these points to the Discussion section.

Comment #14: To follow the previous comment, even though there is variance in the data to whether it is 5 Hz, 10 Hz or broadband changes, each of the findings suggests that there is a disruption in neural activity concomitant with cognitive dysfunction - which is also important to point out.

Response: See response to previous comment.
